# Criticality in probabilistic models of spreading dynamics in brain networks: Epileptic seizures

**DOI:** 10.1371/journal.pcbi.1010852

**Published:** 2023-02-07

**Authors:** S Amin Moosavi, Wilson Truccolo

**Affiliations:** 1 Department of Neuroscience, Brown University, Providence, Rhode Island, United States of America; 2 Carney Institute for Brain Science, Brown University, Providence, Rhode Island, United States of America; Brandeis University, UNITED STATES

## Abstract

The spread of seizures across brain networks is the main impairing factor, often leading to loss-of-consciousness, in people with epilepsy. Despite advances in recording and modeling brain activity, uncovering the nature of seizure spreading dynamics remains an important challenge to understanding and treating pharmacologically resistant epilepsy. To address this challenge, we introduce a new probabilistic model that captures the spreading dynamics in patient-specific complex networks. Network connectivity and interaction time delays between brain areas were estimated from white-matter tractography. The model’s computational tractability allows it to play an important complementary role to more detailed models of seizure dynamics. We illustrate model fitting and predictive performance in the context of patient-specific Epileptor networks. We derive the phase diagram of spread size (order parameter) as a function of brain excitability and global connectivity strength, for different patient-specific networks. Phase diagrams allow the prediction of whether a seizure will spread depending on excitability and connectivity strength. In addition, model simulations predict the temporal order of seizure spread across network nodes. Furthermore, we show that the order parameter can exhibit both discontinuous and continuous (critical) phase transitions as neural excitability and connectivity strength are varied. Existence of a critical point, where response functions and fluctuations in spread size show power-law divergence with respect to control parameters, is supported by mean-field approximations and finite-size scaling analyses. Notably, the critical point separates two distinct regimes of spreading dynamics characterized by unimodal and bimodal spread-size distributions. Our study sheds new light on the nature of phase transitions and fluctuations in seizure spreading dynamics. We expect it to play an important role in the development of closed-loop stimulation approaches for preventing seizure spread in pharmacologically resistant epilepsy. Our findings may also be of interest to related models of spreading dynamics in epidemiology, biology, finance, and statistical physics.

## Author summary

We introduce a new probabilistic model for understanding and predicting the spreading dynamics of epileptic seizures in patient-specific brain networks. The model allows the prediction of whether a seizure will spread or not based on derived phase diagrams that take brain excitability and global network connectivity strength as control parameters. In addition, it also predicts the temporal sequence of spreading across different nodes in patient-specific networks. Importantly, the model’s tractability allows mean-field and finite-size scaling analyses to better understand the nature of the seizure spreading dynamics in large networks, revealing the existence of both discontinuous and continuous (critical) phase transitions. In addition to furthering the understanding of spreading dynamics, these findings are fundamental for the development of new closed-loop control approaches for preventing seizure spread, the main impairing factor leading to loss-of-consciousness, in people with pharmacologically resistant epilepsy.

## Introduction

Epilepsy is one of the most common neurological disorders affecting approximately 65 million people worldwide [1, 2]. About 30% of the cases are diagnosed as pharmacologically resistant epilepsy. Main alternative therapeutic approaches consist of surgical resection of identified epileptogenic brain areas [3, 4] or electrical stimulation [5–8]. In the particular case of focal epilepsy, seizures that initiate in a localized brain region may or may not spread across the brain. While the focal localized seizure onset is commonly not the most impairing aspect, the spreading itself is the main event typically leading to major disruptions in sensorimotor and cognitive processing, as well as loss-of-consciousness. To address the problem of spreading dynamics in epileptic seizures, we introduce a new discrete-state probabilistic network model inspired by continuous-state neural mass Epileptor network models [9]. While complementary to the Epileptor model approach, the probabilistic model allows us here to address fundamental questions about the spreading dynamics which would be otherwise too analytically challenging or computationally intensive.

Data-driven patient-specific Epileptor network models have been widely used to study the dynamics and propagation of focal epileptic seizures [9–12]. In addition to capturing many dynamical properties of seizures in a small brain area, data-driven Epileptor network models [13–17] have been used to predict seizure propagation in patient-specific networks where each node of the network represents a specific brain area. Dynamics of each node is a neural mass model governed by 6 coupled differential equations (Materials and methods). There are three time scales in the model: a fast time scale for high frequency oscillations during a seizure, a slow time scale capturing both interictal and ictal spike-wave discharges, and a very slow time scale for the variable that captures the dynamics of ionic concentrations and metabolic effects that are thought to be responsible for initiation and termination of seizures. In addition to the above three intrinsic time scales, one can consider an additional refractory period after seizure termination (postictal period).

The connectivity matrices for patient-specific networks are obtained by white-matter brain tractography (structural connectivity) in patients with pharmacologicaly resistant epilepsy. Seizures in the network initiate in specific nodes known as epileptogenic zones (EZs) with high intrinsic excitability where seizures can spontaneously initiate. Beyond its dependence on excitability, seizure initiation in an EZ node depends also on its interactions with non-EZ nodes through diffusive couplings. As a result seizure initiation in the EZ node can be, in some cases, inhibited by non-EZ nodes in a non-seizure state. After initiation in the EZ node, a seizure can spread to the other nodes via the network connectivity, thus affecting the very slow variable of surround target areas through diffusive coupling (Materials and methods). Depending on the excitability level and connection strengths, the target node may or may not be recruited into the seizure dynamics. These dynamics have been shown to capture the qualitative features of seizure spread in patients with epilepsy [13, 14].

Analysis of phase diagrams for seizure spread in patient-specific Epileptor networks, as well as their prediction based on local linear stability analyses, have been examined in [18]. In the control parameter space of excitability and global network coupling strength, three phases are observed: a phase in which the EZ node is inhibited such that not even a seizure in the EZ node is possible (*no-seizure*), a phase in which a seizure spontaneously initiates in the EZ node but it does not spread (*no-spread*), and a phase in which the seizure is initiated in the EZ node and it does spread partially or fully (*spread*) to the surrounding network.

Importantly, as we show in this study, large fluctuations in seizure spread are observed near the transition regions from no-spread to spread phases suggesting critical behavior in the spreading dynamics. However, due to the small size of available patient-specific networks, difficulties in obtaining analytical results, as well as the prohibitive computational cost of simulating very-large Epileptor models on general networks, the nature of these phase transitions and potential critical properties remains unclear. For instance, it is not clear how the size of fluctuations and network responses behave as a function of network size, perturbations in neural excitability and global connectivity strength, or external inputs. The hallmark of criticality, i.e. continuous phase transitions, is the divergence of fluctuation sizes and response functions at the critical point, as well as power-law scaling near the critical point in both equilibrium and non-equilibrium systems [19–23].

As stated earlier, the problem is crucial not only to furthering the understanding of the basic neuroscience of epileptic seizures, but also to the ongoing development of approaches for prevention and control of pharmacologically resistant seizures, such as closed-loop intracranial electrical stimulation via the NeuroPace RNS system. This application context suggests also several choices and constraints to our model development. Given the knowledge that a seizure has just started and remains localized, one would like to determine the optimal intervention (spatiotemporal stimulation) in terms of perturbations of network excitability, connectivity strength, or other related network properties to prevent seizure spreading. Here, we also focus only on typical seizures that self-terminate, i.e. we do not consider the case of status-epilepticus.

To determine the nature of these phase transitions in the above application context, we introduce here a probabilistic trinary network model for seizure spread. Furthermore, we show that the model can be easily fitted to time series data and serve as a computationally efficient alternative for predicting seizure spreading dynamics. Regarding the model fitting, because we find analytic forms for phase diagram boundaries, the model fitting largely reduces to simple curve fitting. Three dynamical states are considered for each network node: *susceptible to seizure* (preictal), *active* (seizure, ictal) and *postical refractory*. State dependent conditional probabilities are defined for transitions between these three states. In contrast with the Epileptor network model, here we are not concerned with the details of the fast dynamics in a node. Instead, we consider only two slow timescales. A time scale for the duration of seizures in a node and a refractory (postictal) timescale. While the three states of the model are reminiscent of common Susceptible-Infected-Recovered-Susceptible (SIRS) epidemiological models [24, 25], its dynamics is motivated by that of seizure spread in neuronal networks. In addition, while commonly used SIRS and related models are Markov, the proposed model includes history effects and interaction time delays.

In this study, we focus only on the spreading dynamics within a seizure (our main application context of spread prevention), followed by a refractory or postictal period without transitioning back into susceptible states as in SIRS models. Nevertheless, the model can also be easily adapted to include the inter-seizure dynamics. Because the time intervals between seizures commonly consist of hours if not days, brain areas that have seized will have already roughly recovered to susceptible states (instead of still remaining in the postictal refractory state) before the next seizure. As a consequence, every seizure starts anew in our simulations of the proposed probabilistic model. The initial conditions are always the same: all nodes start in the normal susceptible state, with the surrounding nodes in a non-pathological excitability level, and the EZ area in a hyperexcitable state. Furthermore, parameters such as global excitability, EZ excitability, and coupling strength are assumed to be fixed during a seizure, but are allowed to vary at slower time scales, i.e. across different stochastic seizure realizations. Similarly to nonequilibrium phase transitions in models of directed percolation [20], seizure spreading dynamics in the proposed probabilistic model, under the above specific setup, can have a very large number of absorbing states across different seizure simulations: any spread configuration where a subset of nodes goes into the postictal refractory period.

We show that despite its simplicity, the proposed probabilistic model not only captures seizure spread dynamics and its three phases but also predicts the temporal order of seizure recruitment observed in patient-specific Epileptor network models. In addition, the simplicity and probabilistic nature of the model allow mean-field analysis as well as large-scale computer simulations. Using mean-field approximations and finite-size scaling analyses, we show that transitions from the no-spread to the spread phase include both discontinuous and continuous (critical) non-equilibrium phase transitions, akin to first- and second-order transitions in equilibrium systems, respectively [19]. Furthermore, we show that both the size of fluctuations and response functions diverge at a critical point in the thermodynamic limit. Notably, the size of fluctuations and response functions near the critical point exhibit power-law behavior. At this critical point, the distribution of seizure spread sizes transitions from unimodal to bimodal. These results are supported by numerical simulations of the model on large-scale random networks, and comparisons with Epileptor and proposed probabilistic network models endowed with patient-specific network connectivity.

## Results

### The model

We model the dynamics of seizure spread in a network of interacting nodes. In the initial following sections, we focus on patient-specific finite-size networks characterized by patient-specific connectivity matrices *W*, the corresponding interaction time delay matrices *τ*, and a set of patient-specific epileptogenic and non-epileptogenic zones (EZ and non-EZ nodes) identified by the clinical team involved in the collection of these data (Materials and Methods, and Fig A and Table A in S1 Text). Specifically, the interaction from node *j* to *i* is specified by the interaction weight *w* × *W*_*ij*_ ≥ 0, where *w* is the global connectivity strength, *W*_*ij*_ is the normalized connectivity weight from node *j* to *i*, and *W*_*ii*_ = 0. Due to the fact that we get the *W* matrix from white-matter tractography *W*_*ij*_ ≥ 0. In addition, we consider time delays *τ*_*ij*_ resulting from axonal conduction delays and synaptic dynamics. Each node *i* is assigned a dynamical trinary variable *x*_*i*_(*t*) ∈ {−1, +1, 0}, where the three values correspond to the three possible states of being susceptible to seizure *x*_*i*_ = −1, active (seizure, ictal) *x*_*i*_ = 1, and the postictal refractory state *x*_*i*_ = 0.

The network system is composed of two types of nodes: EZ and non-EZ nodes. EZ nodes can undergo spontaneous transitions into seizure states, while non-EZ nodes are stable unless their interaction with an active EZ node brings them also into a seizure state. This difference between EZ and non-EZ nodes is reflected in their different neural excitability *E*_*j*_. We assign an *E*_*j*_ to each node *j* depending whether the node is an EZ or not, specifically

$$\begin{array}{c} \text{EZ}=\{j\in \left\{1,2,\ldots ,N\right\}|E_{j}>0\} \end{array}$$ (1)

$$\begin{array}{c} \text{non}-\text{EZ}=\{j\in \left\{1,2,\ldots ,N\right\}|E_{j}\leq 0\}. \end{array}$$ (2)

Here we use homogeneous excitability for the non-EZ nodes, i.e. *E*_*j*_ = *E* for all non-EZ nodes. Similarly, for the EZ nodes we set *E*_*j*_ = *E*_*ez*_.

In the following, we introduce the stochastic dynamics of the proposed probabilistic network model in discrete time, specified by the corresponding transition rate functions in continuous time. This choice is motivated by our later use of this discrete-time representation in a mean-field approximation of the model’s dynamics. Nevertheless, the simulations of the probabilistic network model itself and the direct derivations of its phase diagrams are based on the continuous-time dynamics given by the rate functions. For a small enough time interval Δ, the stochastic dynamics are governed by the following conditional transition probabilities

$$\begin{array}{cc} & P(x_{i}=1\;\text{in}\;\left(t,t+\Delta \right]|x_{i}\left(t\right)=-1)=f\left(z_{i}\left(t\right)+E_{i}\right)\Delta +o\left(\Delta \right) \end{array}$$ (3)

$$\begin{array}{cc} & P(x_{i}=0\;\text{in}\;\left(t,t+\Delta \right]|x_{i}\left(t\right)=1)=g\left(t-t_{so,i},{\tilde{\tau }}_{s,i},q_{s,i}\right)\Delta +o\left(\Delta \right) \end{array}$$ (4)

$$\begin{array}{cc} & P(x_{i}=-1\;\text{in}\;\left(t,t+\Delta \right]|x_{i}\left(t\right)=0)=g\left(t-t_{sf,i},{\tilde{\tau }}_{r,i},q_{r,i}\right)\Delta +o\left(\Delta \right), \end{array}$$ (5)

where *f* and *g* are rate functions defined below. In words, Eq 3 is the probability of a node *i* transitioning from susceptible to the active (seizure) state in the time interval (*t*, *t* + Δ]. Similarly, Eq 4 is the transition probability from the seizure state to the postical refractory state, and Eq 5 accounts for the transition from the postictal refractory state to the susceptible state.

The rate function *f* is given by

$$\begin{array}{c} f\left(y\right)=r\times \{\begin{array}{cc} 0 & y\leq 0\\ y & 0\leq y\leq 1\\ 1 & y\geq 1, \end{array} \end{array}$$ (6)

where *r* is a parameter (in per second). In Eq 3, the argument of *f* is the total input *z*_*i*_(*t*) from the network to node *i* plus the excitability *E*_*i*_ of that node. If the input *z*_*i*_(*t*) to a susceptible node *i* with *x*_*i*_ = −1 is positive and large enough, then there will be a non-zero probability of transition from susceptible to seizure state. Specifically,

$$\begin{array}{cc} z_{i}\left(t\right) & =wa\sum_{j}W_{ij}u_{j}\left(t-\tau_{ij}\right)+wb\sum_{j\notin \text{EZ}}W_{ij}E_{j}v_{j}\left(t-\tau_{ij}\right) \end{array}$$ (7)

$$\begin{array}{cc} u_{j}\left(t\right) & =\{\begin{array}{cc} \frac{t-t_{so,j}}{\tau_{s}} & t_{so,j}<t<t_{sf,j}\\ \frac{D_{j}+t_{sf,j}-t}{\tau_{s}} & t_{sf,j}<t<D_{j}+t_{sf,j}\\ 0 & \text{otherwise} \end{array} \end{array}$$ (8)

$$\begin{array}{cc} v_{j}\left(t\right) & =\{\begin{array}{cc} 1 & x_{j}\left(t\right)=-1\\ 0 & \text{otherwise}, \end{array} \end{array}$$ (9)

where the term *wW*_*ij*_*u*_*j*_(*t* − *τ*_*ij*_) accounts for the excitatory input from an active node *j* to a node *i* that is accumulated over the seizure duration with a time scale *τ*_*s*_. The term *u*_*j*_(*t*) is formulated so that the accumulated input begins to dissipate with the same time scale after seizure termination. The terms *t*_*so*, *j*_ and *t*_*sf*, *j*_ are respectively the seizure onset and offset times in node *j* and *D*_*j*_ = *t*_*sf*,*j*_ − *t*_*so*,*j*_ is the duration of seizure in node *j*. The effect of a susceptible node *j* on node *i* is conveyed by the term *wW*_*ij*_*E*_*j*_*v*_*j*_(*t* − *τ*_*ij*_). Recall the excitability *E*_*j*_ is negative for non-EZ nodes and reflects an inhibitory effect. In this way, this term incorporates the diffusive coupling effects featured in the Epileptor model (Materials and methods). The parameters *a* ≥ 0 and *b* ≥ 0 account, respectively, for the strength of the two right terms contributing to *z*_*i*_(*t*) in Eq 7.

In the transition probability from seizure to refractory state, Eq 4, the rate function *g* is defined as

$$\begin{array}{c} g\left(T,\tau ,q\right)=\{\begin{array}{cc} 0 & T\leq \tau -q\\ \frac{1}{q+\tau -T} & \tau -q<T<\tau +q, \end{array} \end{array}$$ (10)

where ${\tilde{\tau }}_{s,i}$ accounts for the time-scale of seizure duration in node *i* and *q*_*s*, *i*_ specifies the variability of seizure duration. The form of the function *g* guarantees that seizure duration *D*_*i*_ in a node *i* falls in the range ${\tilde{\tau }}_{s,i}-q_{s,i}<D_{i}<{\tilde{\tau }}_{s,i}+q_{s,i}$. That is because, if the time since seizure initiation in node *i* is smaller than ${\tilde{\tau }}_{s,i}-q_{s,i}$, the function $g(t-t_{so,i},{\tilde{\tau }}_{s,i},q_{s,i})=0$ (the rate of seizure termination is zero). For a node *i*, this rate is non-zero in the range ${\tilde{\tau }}_{s,i}-q_{s,i}<t-t_{so,i}<{\tilde{\tau }}_{s,i}+q_{s,i}$ and tends to infinity as $t-t_{so,i}\to {\tilde{\tau }}_{s,i}+q_{s,i}$. Therefore, the duration of a seizure in node *i* cannot be larger than ${\tilde{\tau }}_{s,i}+q_{s,i}$. Similarly, for the refractory time *T*_*i*_ in a node *i* we have ${\tilde{\tau }}_{r,i}-q_{r,i}<T_{i}<{\tilde{\tau }}_{r,i}+q_{r,i}$. The variables ${\tilde{\tau }}_{s,i}$, *q*_*s*,*i*_, ${\tilde{\tau }}_{r,i}$, *q*_*r*,*i*_ can generally be functions of the history of the system and interactions. Here, we consider the following forms for ${\tilde{\tau }}_{s,i}$ and *q*_*s*,*i*_.

$$\begin{array}{c} {\tilde{\tau }}_{s,i}=\frac{\tau_{s}}{1-cw\sum_{j\notin \text{EZ}}W_{ij}E_{j}v_{j}\left(t-\tau_{ij}\right)} \end{array}$$ (11)

$$\begin{array}{cc} q_{s,i} & =d{\tilde{\tau }}_{s,i}, \end{array}$$ (12)

where we assume that the seizure termination probability increases with inhibitory inputs, reducing the expected duration of seizure. The inhibitory effect ($cw\sum_{j\notin \text{EZ}}W_{ij}E_{j}v_{j}\left(t-\tau_{ij}\right)$), which appears in the denominator of ${\tilde{\tau }}_{s,i}$, mimics the phenomenon of surround inhibition in focal seizures which is expected to reduce the seizure duration. This inhibitory effect is assumed to be proportional to the interaction weights *wW*_*ij*_ from susceptible nodes which are not in the seizure state. In addition we assume that the variability in seizure duration in each node, specified by *q*_*s*,*i*_, is proportional to seizure duration. The parameters *c* ≥ 0 and *d* ≥ 0 and the function *g* specify the termination probability of seizures. In Fig O in S1 Text we show that the above choices are in agreement with the seizure duration statistics in patient-specific Epileptor network models (See also Materials and methods: Fitting the proposed probabilistic model to patient-specific Epileptor network simulation data).

**Fig 1:**
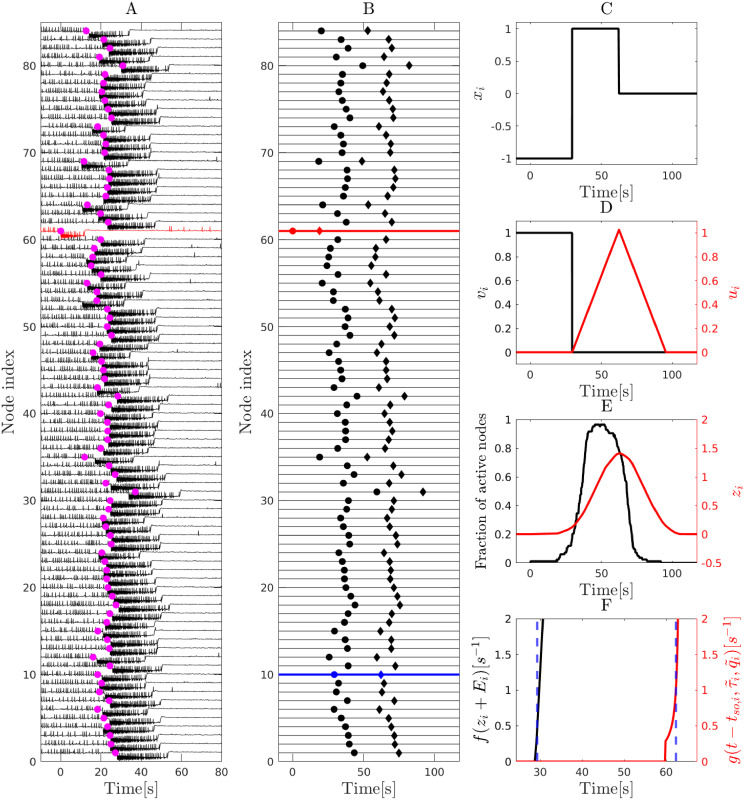
Dynamics of the probabilistic model. An example of seizure spreading dynamics in a patient-specific Epileptor network (subject P1) based on an 84-node parcellation of brain areas (Desikan-Killiany Atlas; see also Materials and methods) and the corresponding dynamics in the proposed probabilistic model. **A** Seizure spread observed in a simulation of a patient-specific Epileptor network model with global coupling strength set to *w* = 0.45, and excitability levels set to *x*_0_ = −2.173 and *x*_0,*ez*_ = −1.8 for the surrounding and epileptogenic nodes, respectively (Materials and methods). The seizure starts at the EZ node (red; node 61; onset time at 0), and then spreads to all other nodes. A postical refractory period follows after seizure termination in each node. (The “spikes” preceding the seizure onset correspond to interictal spikes). Pink dots show rescaled seizure onset times obtained by simulating the proposed probabilistic model shown in panel B. **B** Seizure spread in a simulation of the proposed model. Parameters of the model are *a* = 0.46, *b* = 0.0021, *c* = 1.3, *d* = 0.05, *w* = 0.45, *E* = −0.112, *E*_*ez*_ = 0.0026. Seizure onset and offset times in each node are shown by a circle and a diamond, respectively. Panels **C**-**F** show the evolution of different dynamical variables and transition rates of node 73 (shown in blue in panel B). **C** Evolution of the state variable *x*_*i*_ for *i* = 73. Before seizure onset *x*_*i*_ = −1 (susceptible state); during the seizure *x*_*i*_ = 1 (seizure state) and after seizure termination *x*_*i*_ = 0 (refractory state). **D** Evolution of variables *u*_*i*_ and *v*_*i*_ defined in Eqs 8 and 9. **E** Evolution of the fraction of active (seizing) nodes in the system and *z*_*i*_(*t*) as defined in Eq 7. **F** Evolution of transition rates: *f*(*z*_*i*_ + *E*_*i*_) for transitioning from the susceptible to the seizure state, and $g(t-t_{so},{\tilde{\tau }}_{s,i},{\tilde{q}}_{s,i})$ for transitioning from the seizure to the refractory state. Blue dashed lines indicate seizure onset and offset times. For examples of a small spread size see Figs B and C in S1 Text.

As an illustrative example, we show in Fig 1A and 1B that the proposed probabilistic model qualitatively captures the seizure spread dynamics in a patient-specific Epileptor network. In addition, we show how different dynamical variables *x*_*i*_, *u*_*i*_, *v*_*i*_, transition rates $g(t-t_{so,i},{\tilde{\tau }}_{i},{\tilde{q}}_{i})$ and *f*(*z*_*i*_ + *E*_*i*_) evolve before during and after the seizure for an individual node in the network. (See Figs B and C in S1 Text for more examples in which the seizure spreads only partially).

In the following sections, we show in more detail that the proposed probabilistic model captures the qualitative nature of seizure spreading dynamics in patient-specific Epileptor networks (Materials and methods). We fit the parameters of the model to capture the same spreading dynamics and corresponding phase diagrams as obtained for the Epileptor network model. We also assume the common scenario seen in actual epileptic seizures where there is no recovery from the refractory state while a seizure is still active in the network. To achieve that, the refractory time scale ${\tilde{\tau }}_{r,i}=\tau_{r}$ is fixed for all the nodes and is much longer than the seizure time scale ${\tilde{\tau }}_{s,i}$.

### Phase diagram of the probabilistic model

To derive the phase diagram of the proposed probabilistic model, we considered three main control parameters in the system: the interaction strength *w*, the excitability of the EZ node *E*_*ez*_ and the excitability of non-EZ nodes *E*. Thus, the parameters (*E*_*ez*_, *E*, *w*) did not depend on the network dynamics and were set to fixed values when simulating multiple stochastic realizations corresponding to a given point in the control parameter space for the phase diagrams. Here we consider a network in which we have a single EZ node *ez*. The initial condition for the network is {(∀*i* ∈ {1, 2,.., *N*}), *x*_*i*_(0) = −1} where *N* is the number of nodes in the network. Under this condition, because there is no seizure in the system, all *u*_*i*_(*t*) = 0 and all *v*_*i*_(*t*) = 1. Therefore, using Eq 7 we can simply write *z*_*ez*_(*t*) = *Ewb*∑_*j*_ *W*_*ez*,*j*_. Next, using the rate function for the transition between the susceptible to the seizure state, Eq 6, it is clear that the transition rate *f*(*z*_*ez*_(*t*) + *E*_*ez*_) is nonzero if

$$\begin{array}{c} E_{ez}+z_{ez}\left(t\right)=E_{ez}+Ewb\sum_{j}W_{ez,j}>0. \end{array}$$ (13)

Therefore, for the parameter range in the (*w*, *E*) space in which this inequality holds, the probability of seizure initiation in the EZ node is nonzero and a seizure will eventually start at the EZ. On the other hand, for the parameter range in which the above inequality does not hold, despite having *E*_*ez*_ > 0, the EZ node is inhibited by the susceptible nodes in the network (surround inhibition) that leads to prevention of spontaneous seizures.

Next, we focus on the probability of seizure spread from EZ to non-EZ susceptible nodes. Based again on Eq 6 and assuming that the EZ node is in the seizure state *x*_*ez*_ = 1 and that the rest of the network is in the susceptible state *x_i_*_∉EZ_ = −1, we calculated the transition rate of a susceptible node *i* going to seizure as *f*(*z*_*i*_(*t*) + *E*), where

$$\begin{array}{c} z_{i}\left(t\right)=u_{ez}\left(t\right)W_{i,ez}+wbE\sum_{j\notin \text{EZ}}W_{ij}. \end{array}$$ (14)

It is obvious that *z*_*i*_(*t*) + *E* > 0 leads to a nonzero probability of excitation for node *i*. Due to the piecewise linear nature of *f*(*y*), node *i* with highest value of *z*_*i*_(*t*) has the largest probability of excitation. Sitting at the boundary of seizure spread to non-EZ nodes, this node will, most probably, be the first that goes to seizure after the EZ node. Considering the fact that the term *u*_*ez*_(*t*) is at its maximum when the seizure is about to end in the EZ node, the spread is expected to happen if

$$\begin{array}{c} E+waW_{i,ez}\frac{D_{ez}}{\tau_{s}}+wbE\sum_{j\notin \text{EZ}}W_{ij}>0. \end{array}$$ (15)

In the above equation *D*_*ez*_ is the duration of seizure in the EZ node whose value affects the phase boundary.

**Fig 2:**
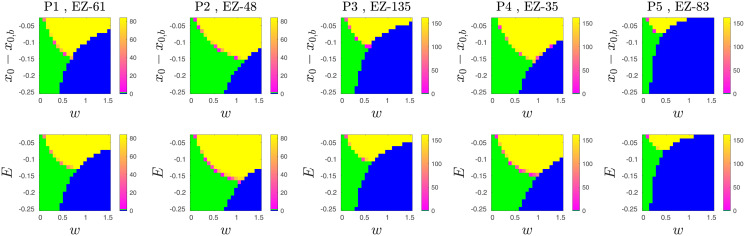
A comparison of the seizure-spread phase diagrams from patient-specific Epileptor networks and proposed probabilistic model. Each panel shows a phase diagram in the space of excitability and global interaction weight. Phase diagrams of the Epileptor network models are shown on the top row for 5 different patient-specific networks. The corresponding diagrams for the probabilistic model are shown on the bottom row. The regions colored as green, yellow, and blue correspond to the phases no-spread (i.e. the seizure remains localized to the epileptogenic zone), spread, and no-seizure (i.e. even the epileptogenic zone does not go into seizure, indicating a strong restrain effect of the surrounding nodes). The colorbar indicates the average spread size across stochastic realizations. The yellow region consists mostly of full spread cases, as illustrated in in Fig 1A and 1B by the time series from both the Epileptor network and probabilistic model network models. Cases of partial small spread are observed closer to the phase boundaries. Note that, while the horizontal axes of top and bottom panels are the same, we set the vertical axes of top panels to be centered at *x*_0,*b*_ for the phase diagrams of Epileptor model in the top row to be comparable with those of the proposed probabilistic model in the bottom row.

The intersection of conditions in Eqs 13 and 15 specifies the parameter range over which there is a nonzero probability of seizure initiation in EZ and a nonzero probability of spread (yellow areas in Fig 2). Additionally, we can specify the parameter range over which there is a nonzero probability of seizure initiation in the EZ node and zero probability of spread (green areas in Fig 2). In this parameter range, the condition in Eq 13 is satisfied, but the condition in Eq 15 is not. Also, there is a parameter range over which the condition in Eq 13 is not satisfied, and regardless of condition in Eq 15, there is no chance of seizure (blue areas in Fig 2). So, we identified three phases of seizure activity in the system: spread, no-spread and no-seizure. Using Eq 13, the boundary between no-seizure and seizure in the EZ node can be evaluated by finding the points in the (*w*, *E*) space bellow which the EZ is inhibited and the probability of seizure initiation in EZ is zero (*E*_*ez*_ + *z*_*ez*_(*t*) ≤ 0), and above which the probability of seizure initiation in the EZ node is non zero (*E*_*ez*_ + *z*_*ez*_(*t*) > 0). That can be found by setting (*E*_*ez*_ + *z*_*ez*_(*t*) = 0). Similarly, we can find the boundary between spread and no spread phases using Eq 15. The boundaries of these regions are respectively

$$\begin{array}{c} E=\frac{-E_{ez}}{wb\sum_{j}W_{ez,j}} \end{array}$$ (16)

for the boundary of no-seizure phase, and

$$\begin{array}{c} E=\frac{-waW_{i,ez}D_{ez}}{\tau_{s}\left[1+wb\sum_{j\notin \text{EZ}}W_{ij}\right]}. \end{array}$$ (17)

for the boundary between spread and no-spread phases. Note that in the above equation *i* must be the node with maximum *z*_*i*_(*t*) at *t* = *t*_*so*,*ez*_ + *D*_*ez*_.

### The probabilistic model captures the coarse spreading dynamics in patient-specific Epileptor network models

Here we show that, despite its simplicity, the proposed probabilistic model can capture the seizure spread dynamics of patient-specific Epileptor network models. We first fitted the parameters of the model including (*a*, *b*, *c*, *d*, *τ*_*s*_) to time series (summarized as spread size and its temporal evolution) of simulated Epileptor networks. We simulated the Epileptor network model (Materials and methods) on 5 different patient-specific networks with two different EZ nodes per network and two different EZ excitability levels *x*_0,*ez*_ ∈ {−1.8, −1.6}. (See Materials and methods, and Fig A and Table A in S1 Text for details on patient-specific connectivity matrices and time delays.) In total, we ran 20 different settings. In addition, for each setting, we also varied a range of global coupling *w* and non-EZ excitability *x*_0_ with 20 stochastic realizations per point in the (*w*, *x*_0_) grid space (Fig 2, top row).

We emphasize that we fitted just a single model or set of parameters to the data from all of the simulated patient-specific Epileptor networks and their parameter variations. To fit the parameters *τ*_*s*_, *c*, *d*, we examined the seizure duration in the EZ node when there was no seizure spread (green area in Fig 2). In this region of the phase diagram the inhibitory input to the EZ node is constant over time, thus ${\tilde{\tau }}_{s,ez}$ is independent of time. In this case, we can calculate the probability distribution of seizure duration in the EZ node as a uniform distribution with mean ${\tilde{\tau }}_{s,ez}$ and standard deviation $q_{s,ez}/\sqrt{3}$ (see Materials and methods for more details). Using maximum likelihood estimation, we fitted the parameters $c=1.3,\tau_{s}=32.22\;s,d=0.05$.

To fit the remaining parameters in the model, we obtained two different data sets from the simulations of Epileptor network models: (a) the boundary of the no-seizure phase (i.e. the boundary of the blue area in Fig 2), and (b) the boundary between the no-spread and spread phases. Eqs 16 and 17 refer to (a) and (b) above, respectively, and can be used to fit the parameters to the Epileptor network data.

Before fitting the two curves we have also to specify the relation between excitabilities in the Epileptor network model (*x*_0_, *x*_0,*ez*_) and the model (*E*, *E*_*ez*_). Considering the excitability level, a single Epileptor network node exhibits a bifurcation point at *x*_0,*b*_ = −2.061. If *x*_0_ > *x*_0,*b*_, the node is an EZ and can undergo spontaneous seizures; if *x*_0_ < *x*_0,*b*_, the node is a non-EZ. Our first choice for the relation between the excitability in the proposed model and Epileptor networks is a proportionality (*E* ∼ *x*_0_ − *x*_0,*b*_ and *E*_*ez*_ ∼ *x*_0,*ez*_ − *x*_0,*b*_). Here we assume

$$\begin{array}{c} E=x_{0}-x_{0,b} \end{array}$$ (18)

$$\begin{array}{c} E_{ez}=(x_{0,ez}-x_{0,b})h, \end{array}$$ (19)

where *h* > 0 is a parameter to be fitted. Using least-squares, we first fitted Eq 17 obtaining the parameters *a* = 0.46, *b* = 0.0021. Finally, we fitted Eq 16 obtaining the parameter *h* = 0.01 (see Materials and methods for more details).

Using an adaptation of the time-rescaling theorem for point processes [26, 27], also known as the temporal Gillespie algorithm [28], we simulated in continuous-time the model with the fitted different parameter sets. The model was simulated with the same patient-specific networks and the same range of parameters (*w*, *E* = *x*_0_ − *x*_0,*b*_) as for patient-specific Epileptor network simulations. Based on these simulations, we can show that the proposed probabilistic model captures well the seizure-spread phase diagrams of Epileptor networks for different patient-specific network connectivity and different EZ spatial locations (Fig 2 and Fig D in S1 Text).

We note that our simulations of the probabilistic models with the temporal Gillespie algorithm are several orders of magnitude faster than the simulation of the corresponding Epileptor network models. This difference is already clear in the amount of calls to random number generators. While simulation of each realization of an Epileptor network requires two calls to random number generators per node per time step in the numerical solution of corresponding stochastic differential equations, simulation of the proposed model in continuous time via the temporal Gillespie algorithm requires only two calls per state transition in the entire network. For concreteness, the simulation of an 84-node Epileptor network during a 120-second simulation, using a simulation step size of 0.001 s (i.e. *M* = 120, 000 steps), results in *M* × *N* = 1.008 × 10^7^ calls to random number generators. In contrast, since a realization of the probabilistic model in continuous time requires only two calls per state transition, the simulation time depends only on the number of transitions between the three states of the model and the number of nodes. For a full seizure spread of size *N* = 84 we have 2 × *N* transitions, and thus a much smaller total of 4 × *N* = 336 calls to random number generators.

**Fig 3:**
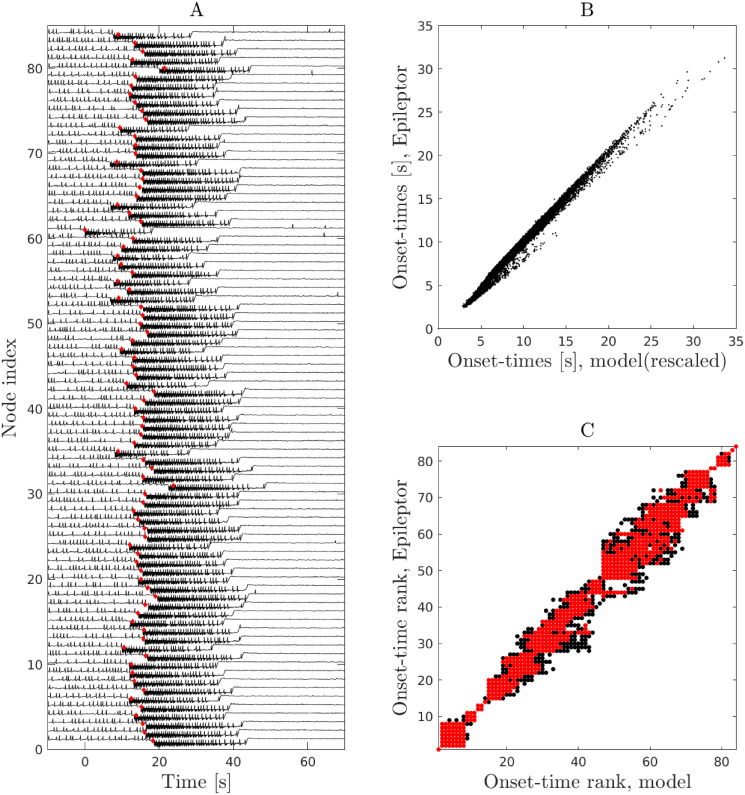
The proposed probabilistic model captures the spread timing in patient-specific Epileptor network models. The plots show the results for patient-specific network P1, EZ-61. **A** A stochastic realization of an Epileptor network simulation in this patient-specific network. The vertical axis specifies the node index in the patient-specific network while the horizontal axis is time centered at the seizure onset time in the EZ node (node 61). Red diamonds specify the expected seizure onset time predicted by the model. **B** Linear relation between mean seizure onset times in Epileptor networks versus the mean onset time in the model for all points in phase space in which we observe full spread (yellow area in Fig 2). We note that, while a linear relation between the seizure onset times is observed for all points in phase space, the slope of the line varies depending on *w* and *E*. We rescaled all the lines to align with the diagonal. **C** Seizure-onset ordering in Epileptor networks versus the proposed model for all points in phase space in which we observe full spread. Red dots specify the 95 percentile of the data.

**Fig 4:**
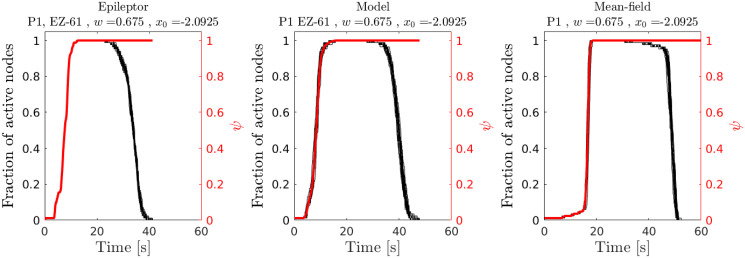
Temporal evolution of average spread size and fraction of active (seizing) nodes: Example for patient-specific network P1, EZ = 61. Each curve contains data from 20 different stochastic realizations. The right vertical axis (red) shows the mean normalized spread size *ψ* = 〈*s*/*N*〉, where *s* is the number of nodes in the surround to which a seizure has spread to. The left panel corresponds to simulations of the patient-specific epileptor network model. The middle panel relates to the simulations of the proposed probabilistic model with the temporal Gillespie algorithm. The right panel shows simulations based on a derived mean-field dynamics (see below and Materials and methods, Mean-field dynamics). In this mean-field approximation, derived under the assumption of Erdős-Rényi random network connectivity, we used an average connectivity weight and an average interaction delay. For this figure, both were computed from the corresponding patient-specific network.

Furthermore, the proposed model closely captures the temporal dynamics of seizure spread in patient-specific Epileptor networks (Fig 3). Seizure onset times in the proposed model are linearly related to those of Epileptor networks with a proportionality factor that is different for different points in the phase diagram. As a result of this linear relationship, the order or rank of seizure recruitment in patient-specific Epileptor networks is also well predicted. In addition, the temporal evolution of the fraction of active (seizing) nodes in the network agrees well with the dynamics in the patient-specific Epileptor network models (Fig 4). The above findings generalized over different patient-specific connectivity networks and EZ locations (Figs E-N in S1 Text).

**Fig 5:**
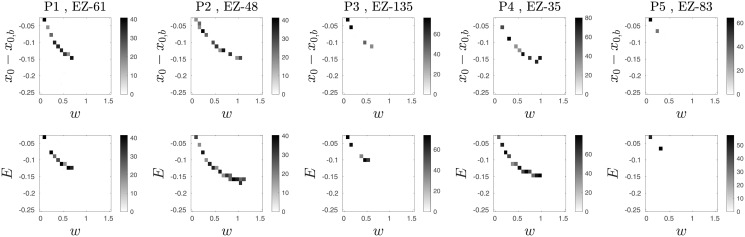
The proposed probabilistic model captures well the size of the fluctuations (standard deviation) in spread size across different stochastic realizations observed at the transition boundary between no-spread and spread phases. In each panel the gray scale colorbar shows the standard deviation of seizure spread across 30 different realizations. The axis of each panel follows the same convention as for the axis in Fig 2. The top row panels are for patient-specific Epileptor network simulations and the bottom row for the probabilistic model.

Importantly, as for Epileptor networks, large fluctuations in spread size are also observed for the proposed model near the boundary between no-spread and spread phases (Fig 5). Studying these fluctuations and their origin is the main goal of the remaining sections of the manuscript.

High fluctuations of this type can appear in near-criticality regions, i.e. near a critical point showing a continuous phase transition. The study of this type of phase transition via numerical simulations is typically computationally very expensive. To overcome this problem, in the next sections we used mean-field approximations to better understand the properties of the phase transition from the no-spread to spread phases.

### Random networks and mean-field phase diagram

A mean-field approximation is expected to predict well the dynamics of the probabilistic model on large enough random Erdős-Rényi (ER) networks. In ER networks the connection probability between any two nodes is *p*. The interaction weight between two nodes is *wW*_*ij*_, where *W*_*ij*_ is a random number obtained by sampling from a uniform distribution with mean *μ*_0_/*N* and standard deviation *σ*_0_/*N*. Division by *N* is necessary for the total interaction strength in the system to be consistent across all network sizes. As a result, the average interaction weight per connection will be *wμ*_0_/*N* and the standard deviation *wσ*_0_/*N*. Further, for convenience, we set the uniform distribution of *W*_*ij*_ to be in the range [0.9, 1.1] × (128/*N*), such that its mean is 1 for *N* = 128. Interaction delays are also uniformly distributed in the range [0.75, 1]/60 seconds. In what follows, we set *p* = 0.2.

We first verify that the mean-field assumption is satisfied for this class of networks in the thermodynamic limit. To be clearer, assume that we have a fraction *ϵ* of the network as EZ nodes in the system. The average input weight to a non-EZ node *i* from all the EZ nodes is

$$\begin{array}{c} w\sum_{j\in \text{EZ}}W_{ij}\approx wpN\epsilon \mu_{0}/N=wp\epsilon \mu_{0}. \end{array}$$ (20)

The law of large numbers guarantees that the approximation becomes exact in the thermodynamic limit *N* → ∞ for constant *p* > 0 and *ϵ* > 0. As *N* → ∞, the mean input converges to a constant value *wpϵμ*_0_ and its standard deviation converges to zero with $∼1/\sqrt{N}$.

To calculate the phase diagrams in the mean-field approximation, we first generalized Eqs 16 and 17, which were derived for the case where there is only one EZ node in the network, to networks with multiple EZ nodes. For the boundary of no-seizure phase, a generalization of Eq 16 requires that we consider multiple EZ nodes. Since all the EZ nodes have the same level of excitability *E*_*ez*_, the only difference between them is the amount of inhibition that they receive from the susceptible nodes. The EZ node with the smallest inhibitory input is expected to go to seizure prior to the others. Therefore for the boundary of no-spread phase we get

$$\begin{array}{c} E_{}=\frac{-E_{ez}}{wb\min_{i}\left\{\sum_{j\notin \text{EZ}}W_{i,j}|i\in \text{EZ}\right\}} \end{array}$$ (21)

where $\min_{i}\left\{\sum_{j\notin \text{EZ}}W_{i,j}|i\in \text{EZ}\right\}$ reflects the smallest surround inhibition among all the EZ nodes. Similarly, the boundary between no-spread and spread phases is evaluated under the assumption that all the EZ nodes are in the seizure state and provide excitatory input to other nodes. At the same time each node also receives inhibitory input from the surrounding nodes. Combining both excitatory and inhibitory inputs, the node with largest *z*_*i*_(*t*) is the one that specifies the boundary between no-spread and spread. As a result for this boundary we get

$$\begin{array}{c} E=-\frac{wa\max_{t}\left\{\sum_{j\in \text{EZ}}W_{ij}u_{j}\left(t-\tau_{i,j}\right)\right\}}{1+wb\sum_{j\notin \text{EZ}}W_{ij}}, \end{array}$$ (22)

where the maximization is with respect to time. In the case of just one EZ node (Eq 17), the maximum happens at the time of seizure termination in the EZ node, while in the case of multiple EZ nodes that maximum value can happen at a different time because of different seizure durations. We emphasise that, similar to Eq 17, the index *i* in this equation indicates the most susceptible node, i.e. the non-EZ node with the highest value of *z*_*i*_(*t*).

Under the assumption that all the EZ nodes go to seizure state simultaneously, it is straightforward to write the mean-field approximation of Eqs 21 and 22 as

$$\begin{array}{c} E_{}=\frac{-E_{ez}}{wbp\mu_{0}\left(1-\epsilon \right)} \end{array}$$ (23)

and

$$\begin{array}{c} E=\frac{-wap\mu_{0}\epsilon \left({\tilde{\tau }}_{s,ez}-q_{s,ez}/2\right)}{\tau_{s}\left(1+wbp\mu_{0}\left(1-\epsilon \right)\right)}, \end{array}$$ (24)

respectively. See Materials and methods for detailed derivations.

**Fig 6:**
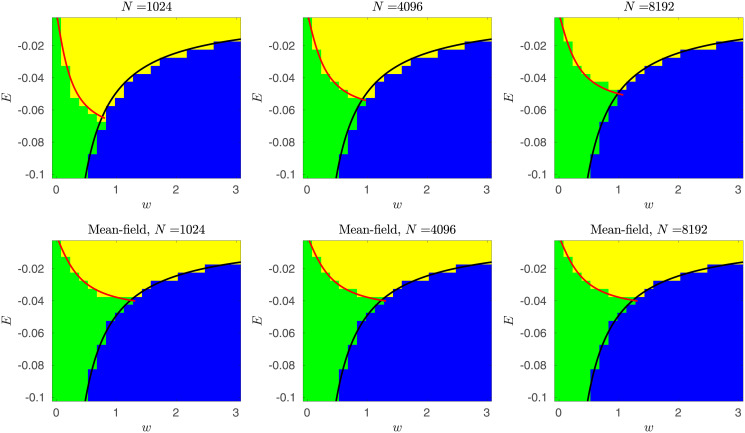
Phase diagrams derived from mean-field approximations. Top row: phase diagrams were obtained from exact continuous time simulations (temporal Gillespie algorithm) of the proposed probabilistic model on ER networks of different sizes *N* = 1024, 4096, 8192. Red curves indicate the boundary between no-spread and spread phases estimated via mean-field finite-size corrected approximations. Black curves denote the boundary separating the no-seizure phase from the other two phases derived from the mean-field approximations. Bottom row: phase diagrams obtained by the simulation of the mean-field dynamics approximation in discrete-time (Eqs 26, 27 and 28; see also Materials and methods). Red and black curves are the transition boundaries derived from the mean-field approximation without the finite-size correction. As the network size grows, the agreement between the two (top and bottom) phase diagrams improves as expected.

Eqs 23 and 24 are expected to specify the exact boundaries of the phase diagram for random networks in the thermodynamic limit. While Eq 23 captures well the behavior of finite-size systems, Eq 24 fails to do so (Fig 6). The failure arises from the fact that finite-size ER random networks exhibit, due to variability in degree distribution and also variability in interaction weights, some degree of heterogeneity. The manifestation of this heterogeneity in the dynamics is higher in small networks with a small number of EZ nodes. As a result, the mean-field approximation fails for such small systems.

To address this shortcoming, we provide a finite-size correction that improves the predictions of phase diagrams. This finite-size correction was obtained by multiplying the right hand side of Eq 24 by the correction factor

$$\begin{array}{c} \nu =1+n\sqrt{\frac{\left(1-\epsilon \right)}{\epsilon pN}}, \end{array}$$ (25)

where *ϵ* denotes the fraction of nodes in the network that belong to the EZ type, *p* is the random network connection probability, and *n* denotes a number to be specified of standard deviations in the number of input links from the EZ nodes to a non-EZ node (see Materials and methods for details).

As expected, the correction term approaches one as *N* → ∞. In Fig 6, we show that with *n* = 2 the finite-size correction captures well the true phase diagrams for different network sizes of *N* ∈ {2^10^, 2^12^, 2^13^}.

### Criticallity in seizure spreading: Analysis based on mean-field dynamics

In order to examine the nature of phase transitions and fluctuations in the probabilistic model of seizure spreading, we developed a mean-field approximation of its dynamics. In this mean-field approach, we worked with a discrete-time approximation. Briefly, we express the network dynamics in terms of transition probabilities for the number of nodes entering and exiting different states at specific discrete times. (See detailed derivations in the Materials and methods: Mean-field dynamics section.)

In the following, given small enough time intervals Δ, we define the following conditional transition probabilities in discrete time for the non-EZ nodes: $P(M_{m+1}|H_{m})$, $P(n_{i,m+1}|H_{m})$, and $P(r_{j,m+1}|H_{m})$. Here, the indices *m*, *i*, *j* denote time bins, and the variables themselves are defined below. The above probabilities can also be similarly defined for the EZ nodes in terms of the corresponding variables $M_{i}^{ez}$, $n_{ij}^{ez}$ and $r_{ij}^{ez}$. All these transition probabilities are conditioned on the history of the process up to and including time bin *m* denoted by

$$\begin{array}{c} H_{m}=\left\{M_{i},n_{ij},r_{ij},M_{i}^{ez},n_{ij}^{ez},r_{ij}^{ez}|i\leq m\;\text{and}\;j\leq i\right\}, \end{array}$$

where *M*_*i*_ and $M_{i}^{ez}$ are respectively the number of non-EZ and the number of EZ nodes that have gone into the seizure state at time bin *i*. The variables *n*_*ij*_ and $n_{ij}^{ez}$ are respectively the number of non-EZ and the number of EZ nodes that have gone into seizure at time bin *i* and have transitioned into the refractory state at time bin *j*. Similarly, the variables *r*_*ij*_ and $r_{ij}^{ez}$ are respectively the number of non-EZ and the number of EZ nodes that have transitioned into the refractory state at time bin *i* and have recovered to susceptible state at time bin *j*.

Specifically, conditioned on $H_{m}$, the probability of having *M*_*m*+1_ non-EZ nodes transitioning to seizure in the time bin *m* + 1 can be written in terms of a binomial distribution

$$\begin{array}{cc} P(M_{m+1}|H_{m}) & =\left(\begin{array}{l} N_{s,m}\\ M_{m+1} \end{array}\right){\left[f\left(z_{m}+E\right)\Delta \right]}^{M_{m+1}}\\ & \times {\left[1-f\left(z_{m}+E\right)\Delta \right]}^{N_{s,m}-M_{m+1}}, \end{array}$$ (26)

where *N*_*s*,*m*_ = *N*_*s*_(*m*Δ) is the number of non-EZ nodes in the susceptible state in the *m*^th^ time bin, and *z*_*m*_ = *z*(*m*Δ) (see Materials and methods for more details).

Similarly, considering *M*_*i*_ non-EZ nodes that have gone into the seizure state at time bin *i* and the history up to time bin *m* ({*n*_*i*,*j*_|*j* ≤ *m*}), we have $M_{i}-\sum_{j=i}^{m}n_{ij}$ out of the *M*_*i*_ nodes that are still in the seizure state. Thus, the conditional probability of having *n*_*i*,*m*+1_ non-EZ nodes (out of the $M_{i}-\sum_{j=i}^{m}n_{ij}$ nodes) transitioning to the refractory state at time bin *m* + 1 is given by

$$\begin{array}{c} P(n_{i,m+1}|H_{m})=\\ \left(\begin{array}{c} M_{i}-\sum_{j=i}^{m}n_{ij}\\ n_{i,m+1} \end{array}\right){\left[g\left(\Delta \left(m-i\right),{\tilde{\tau }}_{s,m},q_{s,m}\right)\Delta \right]}^{n_{i,m+1}}\\ \times {\left[1-g\left(\Delta \left(m-i\right),{\tilde{\tau }}_{s,m},q_{s,m}\right)\Delta \right]}^{M_{i}-\sum_{j=i}^{m+1}n_{ij}}. \end{array}$$ (27)

In the above,

$$\begin{array}{c} {\tilde{\tau }}_{s,m}=\tau_{s}/\left(1-cEw\bar{W}N_{s,m}\right) \end{array}$$

is the mean-field approximation of Eq 11 at time bin *m* and $q_{s,m}=d{\tilde{\tau }}_{s,m}$ is related to the variability of seizure termination times. The term $\bar{W}$ denotes the average interaction weight. It is equal to $\bar{W}=p\mu_{0}/N$ in the case of ER networks as defined above.

Finally, the conditional probability of having *r*_*j*,*m*+1_ non-EZ nodes (out of all the nodes that have transitioned to the refractory state in time bin *j* and are still in refractory state) transitioning from the refractory to the susceptible state at time bin *m* + 1 can be written as

$$\begin{array}{cc} & P(r_{j,m+1}|H_{m})=\\ & \left(\begin{array}{c} \sum_{i=-\infty }^{j}n_{ij}-\sum_{k=j}^{m}r_{jk}\\ r_{j,m+1} \end{array}\right){\left[g\left(\left[m-j\right]\Delta ,{\tilde{\tau }}_{r,m},q_{r,m}\right)\Delta \right]}^{r_{j,m+1}}\\ & \times {\left[1-g\left(\left[m-j\right]\Delta ,{\tilde{\tau }}_{r,m},q_{r,m}\right)\Delta \right]}^{\sum_{i=-\infty }^{j}n_{ij}-\sum_{k=j}^{m+1}r_{jk}}, \end{array}$$ (28)

where ${\tilde{\tau }}_{r,m}=\tau_{r}$ is the refractory time scale and *q*_*r*,*m*_ = *q*_*r*_ specifies the respective variability in the refractory time. As before, because of our particular setup in this study, *r*_*j*,*m*+1_ remains zero throughout the simulations.

As stated above, the above conditional transition probabilities are also similarly defined for the EZ nodes in terms of the variables $M_{i}^{ez}$, $n_{ij}^{ez}$ and $r_{ij}^{ez}$.

Details of the numerical simulation of the above mean-field dynamics are given in the Materials and Methods section. We set $\Delta =\bar{\tau }/m_{\tau }$, where $\bar{\tau }$ is the average interaction delay computed from patient-specific networks and *m*_*τ*_ is the number of simulation time steps in the delay interval $\bar{\tau }$.

We also derived a correction of the above mean-field dynamics for the case of small networks or sparse spread where the number of susceptible nodes is larger than the number of nodes receiving excitation from the seizing nodes (Materials and methods). That was applied to the case of the small patient-specific networks simulated in this study. We verified that these mean-field dynamics capture well the qualitative features of the temporal evolution of seizure spread size and fraction of active (seizing) nodes as in the probabilistic model and Epileptor networks instantiated in these patient-specific networks (Fig 4 and Fig N in S1 Text). Also, in this case of patient-specific networks, the average connectivity weights $\bar{W}$ and the average interaction delays $\bar{\tau }$, used in the mean-field dynamics approximation (Materials and methods), were computed directly from the patient-specific networks. Specifically,

$$\begin{array}{c} \bar{W}=\frac{\sum_{i,j}W_{i,j}}{N(N-1)}, \end{array}$$

and $\bar{\tau }$ as in Eq 48.

**Fig 7:**
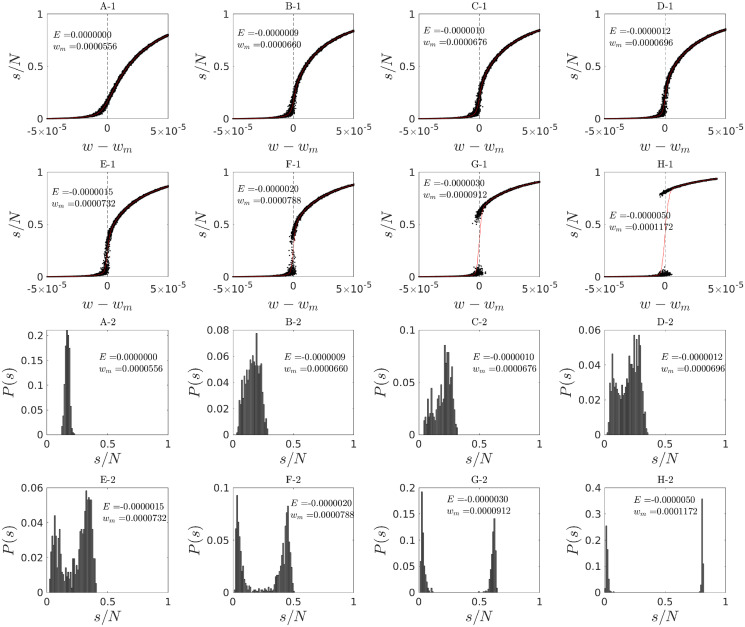
A-1 to **H**-1: **Behavior of the order parameter (normalized spread size) around the point of maximum fluctuations for the mean-field approximation of a system with *N* = 2^15^.** Each plot was obtained by keeping *E* fixed and varying *w* around the point of maximum fluctuations *w*_*m*_ which was obtained by numerical evaluation of the standard deviation of spread sizes. Each point was obtained from the number of nodes recruited to seizure (spread size) in one realization of the mean-field dynamics. For each *w* we plot 50 realizations. Two distinct behaviors are observed: (1) a continuous crossover (without a singularity in the derivatives of the order parameter) for values of *E* close to zero, and (2) a clear discontinuous transition with a jump for *E* < −1.5 × 10^−6^. The shift from continuous to discontinuous behavior is expected to pass through a critical point of a (critical) phase transition. Panels **A**-2 to **H**-2: **Transition between unimodal and bimodal probability distributions of seizure spread size.** Probability distributions of normalized spread size, obtained from 300 to 500 stochastic realizations, are shown at the point of maximum fluctuations *w*_*m*_ and different values of the excitability *E*. A clear transition from unimodal to bimodal distributions is observed. In all simulations *N* = 2^15^. The locations of the above continuous and discontinuous, as well as unimodal and bimodal, regimes in the control parameter space (*w*, *E*) are shown in Fig 8I and 8J with more detail.

**Fig 8:**
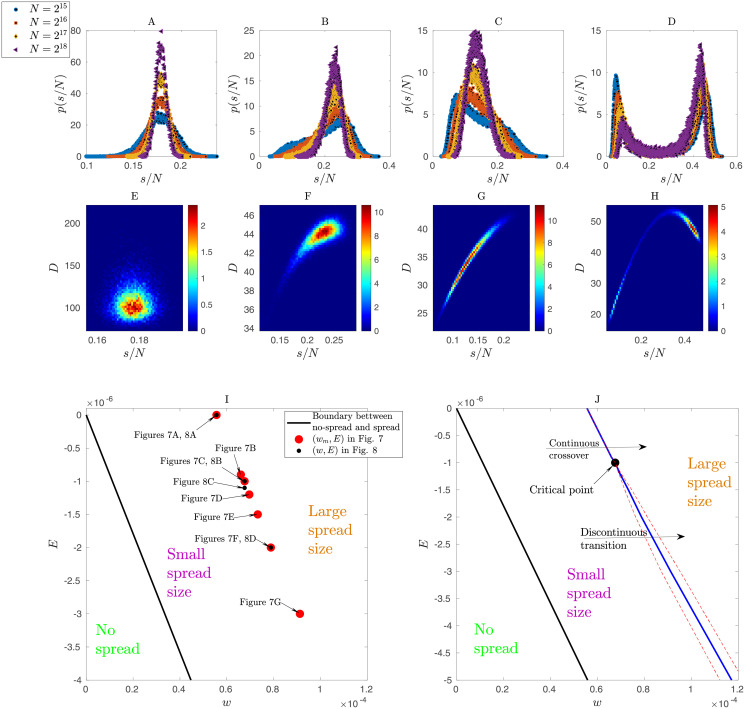
A-D): **Marginal Probability density functions of normalized spread sizes *s*/*N***. Values are shown for different control parameters (*E*, *w*) near the critical point. Presented data were obtained from mean-field simulations of the probabilistic model for different system sizes of *N* = 2^15^, 2^16^, 2^17^, 2^18^. **A** Unimodal distribution in agreement with a Gaussian, observed for a point at the upper part (with respect to the critical point location) of the boundary between no-spread and spread phases (*E* = 0, *w* = 5.5 × 10^−5^). **B,C** Closer to the critical point (*E* = −10^−6^, *w* = 7.67 × 10^−5^ in (B) and *E* = −1.1 × 10^−6^, *w* = 7.67 × 10^−5^ in (C)), the distributions become skewed with large variance. **E** The distributions become bimodal near the lower part of the phase boundary (*E* = −2.00 × 10^−6^, *w* = 7.88 × 10^−5^). (**E-H**): **Joint Probability density functions of normalized spread sizes *s*/*N* and duration of seizures *D*.** Values are plotted as heat maps for control parameters (*E*, *w*) near the critical point. The parameters in panels (E,F,G,H) are respectively the same as in panels (A,B,C,D). Data were obtained from mean-field simulations of the model with system size *N* = 2^18^. **E** Unimodal distribution, which is roughly in agreement with a Gaussian probability density function (but slightly skewed in the duration coordinate), is found on the upper part (with respect to the critical point location) of boundary between no-spread and spread phases. Duration and size of seizures appear to be uncorrelated. **F,G** Near the critical point stronger correlation between spread size and duration of seizures is observed and the distribution exhibits a wider peak and stronger correlation in the two dimensional space of (*D*, *s*/*N*) in G. **H** Moving near the boundary lower to the critical point, the joint distribution becomes bimodal with two distinct modes. The locations of the above unimodal and bimodal regimes in the control parameter space (*w*, *E*) are shown in the next panel with more detail. (**I-J**): **Details of the phase diagram near the critical point.** **I** Parameters are shown in the (*w*, *E*) space. Red dots denote the points at which the variability of spread size across realizations is maximized (*w*_*m*_, *E*) in Fig 7. Black dots denote the points for which we plotted the probability density functions of normalized spread size (*s*/*N*) and the joint probability density of duration (*D*) and spread sizes in Fig.8A-H. **J** The black line indicates the boundary between the no-spread and spread phases of the order parameter. The blue line indicates the location of points of maximum fluctuations in the order parameter. Between the red dashed lines we observe bimodality in the probability distribution of the order parameter. The arrow above the critical point indicates a continuous crossover from small spread to large spread sizes. The arrow below the critical point indicates a transition with a discontinuity in the order parameter, i.e. it is not differentiable at that point. Passing through the critical point results in a continuous transition that is expected to exhibit a singularity in the derivative of the order parameter in the thermodynamic limit. We investigated this expected property via finite-size scaling analysis in Figs 9 and 10. Despite the apparent very small region where the above transition from discontinuous to continuous behavior happens, we emphasize that different choices of parameters and their scaling can constrain the seizure spread activity to this small region. For example, based on Eq 21, we note that a choice of smaller EZ excitability (*E*_*ez*_) level can constrain the spread phase to a very small region around the critical point.

Having derived a mean-field approximation of the spreading dynamics in the proposed model, we proceeded by examining the nature of its phase transitions based on numerical simulations of the mean-field dynamics on ER networks. We examined how the order parameter, i.e. the fraction of spread size *s*/*N* behaves as control parameters vary near the boundary between the no-spread and spread phases in the phase diagram. We simulated many stochastic realizations of the mean-field dynamics on a network of size *N* = 2^15^. Interestingly, we found that the order parameter shows both continuous and discontinuous transitions depending on a specific value of the excitability parameter while varying the global connectivity strength near the phase transition boundary (Fig 7). In particular, there is a clear change of behavior from a continuous to a discontinuous phase transition, suggesting the existence of a critical point. These two different continuous and discontinuous regimes are further manifested in the unimodal and bimodal distributions of seizure spread sizes (Figs 7 and 8). The existence of a transition point between continuous and discontinuous regimes allowed us to approximately locate the critical point.

We examined in more detail the probability distributions of spread sizes via finite-size scaling analyses. The distribution of similar quantities (e.g. avalanche sizes [22]) can show power-law scaling in some, but not all, systems at criticality. Therefore, we checked for the existence of this scaling in the functional form of spread-size distributions near the estimated critical point (Figs 7 and 8). We examined the distributions for increasing network size *N* near the estimated critical point and also at two other points on the boundary between the no-spread and spread phases. Unimodal distributions, found on the upper part (with respect to the critical point) of the phase boundary, were roughly approximated by Gaussian distributions. Near the estimated critical point, the distributions became more and more skewed, but no evidence of power-law behavior was detected (see also Discussion).

**Fig 9:**
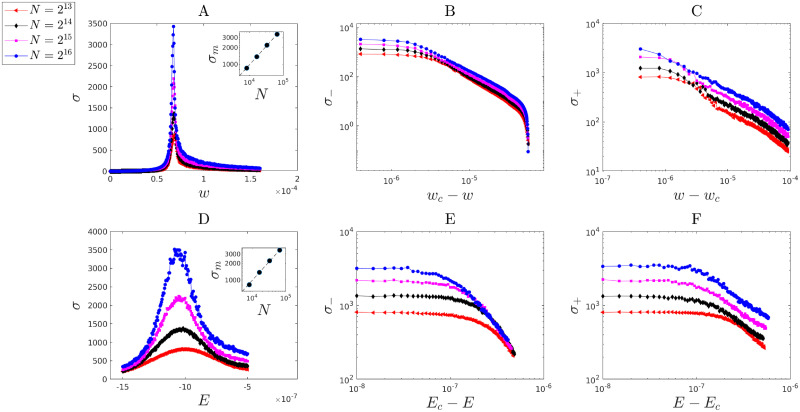
Power-law divergence of stochastic fluctuations in spread size near the critical point. We used finite-size scaling analysis over four different network sizes of 2^13^, 2^14^, 2^15^, 2^16^. **A** The standard-deviation *σ* of the fluctuations as a function of *w* (fixed *E* = *E*_*c*_) near the critical point (*w*_*c*_ ≈ 6.7610^−5^, *E*_*c*_ ≈ 1.0010^−6^). The inset shows the power-law divergence of *σ* at its maximum and the corresponding scaling *σ*_*m*_ ∼ *N*^0.66(1)^. **B,C** Power-law behavior of *σ* shown on log-scale for *w* approaching the critical point from below with corresponding scaling $\sigma_{-}∼{\left(w_{c}-w\right)}^{-\gamma^{\prime }}$ and exponent estimated as ${\hat{\gamma }}^{\prime }=1.63\left(3\right)$, and from above with corresponding scaling *σ*_+_ ∼ (*w* − *w*_*c*_)^−*γ*^ and exponent estimated as $\hat{\gamma }=0.63\left(1\right)$, respectively. **D** The standard-deviation *σ* of the fluctuations as a function of *E* (fixed *w* = *w*_*c*_) near the critical point. The inset shows the power-law divergence of *σ* at its maximum and the corresponding scaling *σ*_*m*_ ∼ *N*^0.68(1)^. **G,H** Power-law behavior of *σ*(*E*) shown on log-scale for *E* approaching the critical point from below with corresponding scaling $\sigma_{-}∼{\left(E_{c}-E\right)}^{-\alpha^{\prime }}$ and exponent estimated as ${\hat{\alpha }}^{\prime }=1.4\left(1\right)$, and from above with corresponding scaling *σ*_+_ ∼ (*E* − *E*_*c*_)^−*α*^ and exponent estimated as $\hat{\alpha }=0.87\left(5\right)$.

Next, we asked the question of how the size of fluctuations behaves near this critical point. The size of fluctuations at a continuous (critical) transition is known [19, 20] to diverge in the thermodynamic limit *N* → ∞. We consider the standard deviation of spread sizes *σ* to assess the size of fluctuations in our model. We show that *σ* appears to diverge at the critical point as a power-law function of excitability and global connectivity strength (Fig 9). The power-law scaling was different on the two sides of the transitions [29]. For fixed *E* = *E*_*c*_, *σ* exhibits power-law behavior as a function of *w* as follows: from above the critical point,

$$\begin{array}{c} \sigma_{+}∼{\left(w-w_{c}\right)}^{-\gamma }, \end{array}$$

and from below as

$$\begin{array}{c} \sigma_{-}∼{\left(w_{c}-w\right)}^{-\gamma^{\prime }}. \end{array}$$

The corresponding estimated exponents are $\hat{\gamma }=0.63\left(1\right)$ and ${\hat{\gamma }}^{\prime }=1.63\left(3\right)$, respectively (Fig 9A–9C).

Similarly, we examined the behavior of *σ* with respect to *E* with fixed *w* = *w*_*c*_. As before, power-law behavior is also observed for *σ* according to

$$\begin{array}{c} \sigma_{+}={\left(E-E_{c}\right)}^{-\alpha } \end{array}$$

and

$$\begin{array}{c} \sigma_{-}={\left(E_{c}-E\right)}^{-\alpha^{\prime }}, \end{array}$$

with estimated exponents $\hat{\alpha }=0.87\left(5\right)$ and ${\hat{\alpha }}^{\prime }=1.4\left(1\right)$, respectively (Fig 9D–9F). We note that the observed power-law domain increases as *N* increases, so that fluctuations at the critical point scale as *σ*_*m*_ ∼ *N*^0.66^.

Furthermore, the response functions with respect to control parameters are also known to diverge at the critical point in the thermodynamic limit. Here, we defined the response functions of the system as

$$\begin{array}{c} \chi_{w}=\frac{\partial \psi }{\partial w} \end{array}$$

and

$$\begin{array}{c} \chi_{E}=\frac{\partial \psi }{\partial E}, \end{array}$$

where *ψ* = 〈*s*/*N*〉.

**Fig 10:**
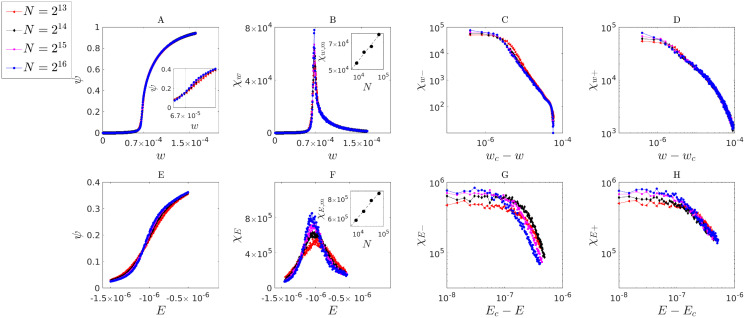
Power-law divergence of response functions $\chi_{w}=\frac{\partial \psi }{\partial w}$ and $\chi_{E}=\frac{\partial \psi }{\partial E}$ near the critical point. We used finite-size scaling analysis over four different network sizes of 2^13^, 2^14^, 2^15^, 2^16^. **A** The expected value of the normalized spread size, *ψ* = 〈*s*/*N*〉, as a function of *w* (fixed *E*) near the critical point (*w*_*c*_ ≈ 6.76 10^−5^, *E*_*c*_ ≈ 1.00 10^−6^). The inset zooms the view around the critical point. **B** The response *χ*_*w*_ plotted as a function of *w* (for fixed *E* = *E*_*c*_). The inset shows the divergence of the maximum response *χ*_*w*,*m*_ as a function of *χ*_*w*,*m*_ ∼ *N*^0.16(1)^. **C,D** The log-scale plots show the power-law behavior of the response function *χ*_*w*_ as *w* approaches the critical point from below with corresponding scaling *χ*_*w*−_ ∼ (*w*_*c*_ − *w*)^*β*′−1^ and exponent estimated as ${\hat{\beta }}^{\prime }=-0.96\left(3\right)$, and from above with corresponding scaling *χ*_*w*+_ ∼ (*w* − *w*_*c*_)^*β*−1^ and exponent estimated as $\hat{\beta }=0.43\left(5\right)$. **E** The expected value of the normalized spread size, as a function of *E* near the critical point (for fixed *w* = *w*_*c*_). **F** The response *χ*_*E*_ plotted as a function of *E* (for fixed *w* = *w*_*c*_). The inset shows the divergence of the maximum response *χ*_*w*,*m*_ as a function of *χ*_*w*,*m*_ ∼ *N*^0.18(1)^. **G,H** The log-scale plots show the power-law behavior of the response *χ*_*E*_ as *E* approaches the critical point from below with corresponding scaling *χ*_*E*−_ ∼ (*E*_*c*_ − *E*)^1/*δ*′−1^ and exponent estimated as ${\hat{\delta }}^{\prime }=-1.6\left(5\right)$, and from above with corresponding scaling *χ*_*E*+_ ∼ (*E* − *E*_*c*_)^1/*δ*−1^ and exponent estimated as $\hat{\delta }=12\left(5\right)$.

Fig 10 shows that both response functions appear to diverge with networks of growing size. The exponents *β*, *β*′ at fixed *E* = *E*_*c*_ were defined here for the behavior of *χ*_*w*_. Approaching the critical point from below leads to

$$\begin{array}{c} \chi_{w-}∼{\left(w_{c}-w\right)}^{\beta^{\prime }-1}, \end{array}$$

and to

$$\begin{array}{c} \chi_{w+}∼{\left(w-w_{c}\right)}^{\beta -1} \end{array}$$

from above. Similarly, we defined the exponents *δ*, *δ*′ for the behavior of *χ*_*E*_, for fixed *w* = *w*_*c*_. The resulting power-law scaling corresponds to

$$\begin{array}{c} \chi_{E-}∼{\left(E_{c}-E\right)}^{1/\delta^{\prime }-1} \end{array}$$

for approaching *E*_*c*_ from below, and to

$$\begin{array}{c} \chi_{E+}∼{\left(E-E_{c}\right)}^{1/\delta -1} \end{array}$$

from above. We defined the exponents so that the behavior of the order parameter *ψ* around the critical point can be written as

$$\begin{array}{c} \psi_{-}=\psi_{c}-\psi \left(w,E=E_{c}\right)∼{\left(w_{c}-w\right)}^{\beta^{\prime }} \end{array}$$ (29)

$$\begin{array}{c} \psi_{+}=\psi \left(w,E=E_{c}\right)-\psi_{c}∼{\left(w-w_{c}\right)}^{\beta } \end{array}$$ (30)

$$\begin{array}{c} \psi_{-}=\psi_{c}-\psi \left(E,w=w_{c}\right)∼{\left(E_{c}-E\right)}^{1/\delta^{\prime }} \end{array}$$ (31)

$$\begin{array}{c} \psi_{+}=\psi \left(E,w=w_{c}\right)-\psi_{c}∼{\left(E-E_{c}\right)}^{1/\delta }. \end{array}$$ (32)

Exponent symbols were chosen in analogy with the standard use in current literature on criticality. There, the exponent *β* typically specifies the relation between the order parameter and control parameters, and the exponent *δ* the relation between the order parameter and an external field. In the standard formulation of critical phenomena, due to the fact that the order parameter is typically zero at the critical point (*ψ*_*c*_ = 0 and *ψ*_−_ = 0), the exponents *β*′ and *δ*′ are not defined. However, our analysis suggests that these exponents exist in the proposed probabilistic model, where power-law behavior is observed around a nonzero value of *ψ* = *ψ*_*c*_ ≈ 0.14.

In other words, the estimated critical point lies inside the region of the defined spread phase. This can happen due to finite-size effects in some models, e.g. kinetic Ising models [30] and epidemic models in the dynamic isotropic percolation universality class [31]. In such models, the estimated *ψ*_*c*_ will decrease and approach zero by increasing system size. Nevertheless, it seems that this is not the case in the proposed probabilistic model because we observe that *ψ*_*c*_ does not decrease by increasing system size. We also note that the bimodal regime occupies a thin region almost parallel to the phase boundary (Fig 8I and 8J). The unimodal regime, with the mode consisting of either a small or large spread, is observed everywhere else inside the spread phase region.

The numeric values of these exponents were estimated as ${\hat{\beta }}^{\prime }=-0.96\left(3\right)$, $\hat{\beta }=0.43\left(5\right)$, ${\hat{\delta }}^{\prime }=-1.6\left(5\right)$, and $\hat{\delta }=12\left(5\right)$. While the exponents $\hat{\beta }$ and $\hat{\delta }$ are positive and in the expected range, the estimated value of ${\hat{\beta }}^{\prime }$ and ${\hat{\delta }}^{\prime }$ are negative. This negative exponent leads to a singularity in *ψ* and that is not acceptable as 0 ≤ *ψ* ≤ 1. We think that these inconsistent exponents result from what has been referred before as apparent exponents [32]. They appear when the scaling function exhibits power-law behavior in such a way that masks the actual critical exponent. (See Materials and methods for a formulation of the behavior of *ψ*_−_ in terms of apparent exponents.)

All of the above estimated exponents are summarized in Table 1. In sum, to our knowledge, the proposed probabilistic model does not belong to any of the well known universality classes. We note, nevertheless, that the estimation of critical points is prone to finite-size effects and numerical inaccuracy. It is possible that more accurate methods may lead to slightly different exponents. We hope these estimated exponents will help to shed some light on the spreading dynamics of epileptic seizures. In particular, the exponents inform about the sensitivity of the modeled spreading dynamics to perturbations in the control parameters (*w*, *E*) and also in external inputs to the system near the critical point.

**Table 1:** Estimated exponents. The terms including *σ* denote the standard deviation (size of fluctuations) of spread size and their dependence on excitability (*E*) and connectivity strength (*w*) in the probabilistic model. The terms including *ψ* relate to the order parameter (normalized spread size) and their dependence on excitability and connectivity strength. The numbers in parentheses after each value represent the error in the last digit, e.g 1.63(3) = 1.63 ± 0.03.

Exponent	Equation	Estimated value
*γ*	$\sigma_{+}{|}_{E=E_{c}}∼{\left(w-w_{c}\right)}^{-\gamma }$	$\hat{\gamma }=0.63\left(1\right)$
*γ*′	$\sigma_{-}{|}_{E=E_{c}}∼{\left(w_{c}-w\right)}^{-\gamma^{\prime }}$	${\hat{\gamma }}^{\prime }=1.63\left(3\right)$
*α*	$\sigma_{+}{|}_{w=w_{c}}∼{\left(E-E_{c}\right)}^{-\alpha }$	$\hat{\alpha }=0.87\left(5\right)$
*α*′	$\sigma_{-}{|}_{w=w_{c}}∼{\left(E_{c}-E\right)}^{-\alpha^{\prime }}$	${\hat{\alpha }}^{\prime }=1.4\left(1\right)$
*β*	$\psi_{+}{|}_{E=E_{c}}∼{\left(w-w_{c}\right)}^{\beta }$	$\hat{\beta }=0.43\left(5\right)$
*β*′	$\psi_{-}{|}_{E=E_{c}}∼{\left(w_{c}-w\right)}^{\beta^{\prime }}$	${\hat{\beta }}^{\prime }=-0.96\left(3\right)$
*δ*	$\psi_{+}{|}_{w=w_{c}}∼{\left(E-E_{c}\right)}^{1/\delta }$	$\hat{\delta }=12\left(5\right)$
*δ*′	$\psi_{-}{|}_{w=w_{c}}∼{\left(E_{c}-E\right)}^{1/\delta^{\prime }}$	${\hat{\delta }}^{\prime }=-1.6\left(5\right)$

As a complementary evidence of criticality, in addition to the above described power-law behavior of response functions, we also found that the maximum values of response functions *χ*_*w*,*m*_, *χ*_*E*,*m*_ tend to diverge with increasing network size *N*. Furthermore, these values also followed power-law functions of *N* according to *χ*_*w*,*m*_ ∼ *N*^0.16(1)^ and *χ*_*E*,*m*_ ∼ *N*^0.18(1)^.

## Discussion

The nature of the spreading dynamics in epileptic seizures remains a challenging problem in neuroscience, with important implications to the development of new therapeutic approaches, especially in the case of pharmacologically resistant seizures. Here, we have provided two novel contributions to address this challenge. First, we have introduced a new probabilistic network model that can capture the spatiotemporal spreading dynamics of seizures in patient-specific complex brain networks. We set up the problem in the context of a focal seizure that has just started and initially remains localized into the epileptogenic zone. The question is then whether the seizure will spread or not, and how. To answer this question, we started by showing that the model can be fitted to data-driven patient-specific Epileptor networks. Because of its probabilistic and phenomenological nature, the model can be easily fitted and is fast to simulate. We then derived the phase diagrams of patient-specific models, where the order parameter was defined as the seizure spread size, and the control parameters were defined as the neural excitability and global connectivity strength. The phase diagrams allowed us to determine whether a seizure will spread based on the excitability and global connectivity strength in the brain. We have also shown that simulations of the model also successfully predicted the temporal evolution of spread size and the temporal ordering or rank of different network nodes in the surrounding as they are recruited into seizure. In this way, fast simulations of the probabilistic model can accurately predict how a seizure spreads.

Second, our analyses revealed the nature of the phase transitions in the seizure spreading dynamics in these probabilistic models. We have demonstrated that the order parameter spread size can show both discontinuous and continuous (critical) phase transitions as neural excitability and global connectivity strength are varied. A mean-field approximation of the dynamics and finite-size scaling analyses provided supporting evidence for the existence of a critical point near the boundary separating the no-spread and spread phases. Specifically, we have shown that the standard deviation of fluctuations in spread size diverges with power-law scaling at numerically estimated critical points as the network size *N* is increased. Furthermore, we have also shown that the corresponding response functions, namely the partial derivatives of the order parameter with respect to excitability and global connectivity strength, also diverge at the critical point with increasing *N*. Importantly, this critical point separates two distinct regimes in the spreading dynamics for control parameters near the boundary between no-spread and spread phases. These two regimes are characterized by either unimodal or bimodal probability distributions of spread size. In the unimodal regime, seizures present spread sizes that range from very small to large number of network nodes in a continuous fashion. On the other hand, in the bimodal regime, seizure spread sizes are typically either very small or very large, with few or no observed intermediate sizes. Stochastic fluctuations trigger either small or these seemingly explosive large spread size events.

We emphasize that currently there is not enough data from recordings in either patients or animal models, both in terms of the number of recorded seizures per subject and in terms of recordings with full coverage of brain areas in both hemispheres. The number of recorded seizures per subject tends to be very small, especially in the hospital setting of epilepsy monitoring units. Available ECoG or SEEG recordings have commonly been restricted to a small subset of brain areas candidate for resective surgery or device implantation. All of this makes the examination of distributions of seizure spread sizes and related statistics based on experimental datasets currently unfeasible or at least very incomplete. Given these data restrictions, here we have adopted patient-specific Epileptor networks as our main reference for the development of the probabilistic model of seizure spreading. As stated earlier, patient-specific Epileptor networks have been fitted to actual patient data and shown to successfully capture many of the seizure dynamics features [13, 14, 17, 33]. In this sense, we think patient-specific Epileptor networks provide an initial good reference for the study of probabilistic models of spreading dynamics in epileptic seizures. Nevertheless, we also note that, given the complexity of Epileptor network models and their costly computational simulations required in finite-size scaling analyses, for example, more concise probabilistic models as the one proposed here can play a fundamental complementary role. We should also emphasize that since the proposed model is a coarsened version of the Epileptor network states, it does not capture all their dynamical features such as synchronization of oscillatory activity across networks nodes and its potential effects on seizure spread. In this sense, we do not claim our analyses elucidate the nature of the phase transitions in the Epileptor model in its entirety. That remains an open question. The presented mean-field analysis is a mean-field analysis of the proposed probabilistic model.

As stated above, our focus in this study has been the scenario more immediately relevant to predicting whether and how a seizure that has just started will spread. In this first treatment, we have ignored how inter-seizure dynamics and potential history effects of spreading patterns in a given past seizure might affect the spreading in future seizures. In other words, we assumed the spreading dynamics in a given seizure to depend only on the within seizure history related to the events that have happened since the seizure initiation and that are independent from spreading events in previous seizures. This might be a reasonable first-order approximation if seizures are sufficiently far apart in time. In terms of model improvement, inter-seizure dynamics can be incorporated via the inclusion of appropriate time scales for the recovery from postictal periods. In addition, recent studies suggest that the temporal dynamics within sequences of seizures show multiple time scales, from circadian to multiday rhythms [34]. Furthermore, once these dynamics are incorporated, one would also need to address the possibility of seizure spread patterns in a given seizure to affect the spreading pattern in future seizures. This might involve synaptic plasticity and maintenance of pathological networks for seizure spread.

Given the choice of parameters and existence of fixed epileptogenic areas that start seizures and drive spreading, and the specific allowed sequences of events to capture typical neural dynamics during seizure onset and spread, the proposed probabilistic model lacks detailed balance, the condition for thermodynamic equilibrium. Therefore, our use of phase transition and related terminology needs to be considered outside the statistical physics of systems in equilibrium. The concept of phase transitions and critical behavior has over the years been extended to systems near and far from equilibrium, e.g. [20, 35]. The main related reference to our study here is the field of directed percolation (DP) and other related models of nonequilibrium systems [20, 36–39]. Although the proposed probabilistic model is more complex and does not belong to the DP universality class, our use of concepts such as phase transitions, criticality, and our power-law scaling analyses are analogous to that commonly performed in the DP and related fields.

We have demonstrated the existence of signatures of criticality based on divergence of fluctuations in spread size and related response functions at estimated critical points. Furthermore, the exponents for power-law scaling differed between the two sides of the transition [29]. However, in contrast to many previous studies focused on distributions of avalanche sizes, our analysis did not find power-law behavior in the functional form of distributions of spread size at the estimated critical points. Nevertheless, we emphasize that, although this is expected in many processes, e.g. avalanches in self-organized critical systems [21, 22], power-law scaling in the probability density of the order parameter (here spread size) can also be absent in many other systems at criticality. Examples of the latter can be found in Ising models where the net magnetization can show bimodal distributions with no power-law scaling [40, 41].

The estimated critical point inside the spread phase region, where fluctuations and response functions appear to diverge, is also at the transition between the two regimes of unimodal and bimodal distributions of spread size in the examined ER random networks. Two main related issues can be raised. First, this transition suggests a bifurcation from a single (small spread) to two metastable states (small and large spread) in the spreading dynamics. One could argue that our finite-size scaling results would be more properly interpreted from the perspective of bifurcation analyses in dynamical systems under stochastic perturbations, rather than from the statistical physics perspective of phase transitions. Nevertheless, these two perspectives are not necessarily mutually exclusive and can be applied in a complementary manner when approaching some nonequilibrium systems, e.g. [35, 42]. Second, it has been shown that in mean-field analyses of certain stochastic nonlinear systems, e.g. stochastic Wilson-Cowan dynamics on random networks, metastability vanishes in the thermodynamic limit *N* → ∞, being replaced by multistability in the resulting deterministic system [43]. In addition, the transition rates between metastable states are expected in this case to decay exponentially with increasing *N*. We did not observe such behavior in our simulations. In contrast, bimodality was enhanced with increasing *N*. Furthermore, we emphasize that in this bimodal regime where stochastic realizations can result in either small or large spread, the occurrence of the later always requires the network to approach first the small spread metastable state. That results, in our setup, from the initial conditions of the network always being the normal susceptible state, i.e. zero spread size. Exponential decay of transition rates would imply that the occurrence of large spread sizes should become less and less likely with increasing *N*, something that we also did not observe. Another potential issue that could be raised is that this bimodal regime could somewhat contribute artifacts to our finite-size power-law scaling analyses. We think that such effects are unlikely given that our finite-size analyses are performed at the estimated critical point and for directions including only the unimodal regime. In sum, although these issues remain open to further investigation, we think that the above arguments support the approach taken here.

While our initial results relating the probabilistic model to Epileptor networks were based on patient-specific complex networks, i.e. networks with modular graphs reflecting the hemispheric and other brain areas organization structures, our mean-field approximations and results relied on the assumption of Erdős-Rényi random networks. Although this disparity did not affect significantly the prediction of phase diagrams and the time evolution of seizure spread size in patient-specific Epileptor networks (Figs 4 and 6, and Fig N in S1 Text), the extension of our response function and finite-size scaling results based on the mean-field approximations to these complex networks remains an important open problem. It also remains to be shown how our results extend to patient-specific networks with much finer parcellation of brain areas and more varied epileptogenic zone locations than those examined in this study. We hope to address these problems in future studies.

We expect that the type of probabilistic models for spreading dynamics proposed here will play a fundamental role in closed-loop intracranial stimulation control approaches for preventing seizure spread. For instance, predictions based on these patient-specific probabilistic models can guide the specification of spatiotemporal stimulation patterns in NeuroPace RNS devices [7, 44] endowed with the ability to track biomarkers of brain excitability and connectivity strength. Finally, we hope that the continuing development of new human neurophysiological recording strategies and devices will soon allow for the experimental testing of the many predictions derived by our analysis of seizure spreading dynamics.

## Materials and methods

### The Epileptor network model

We follow closely the formulation in [15, 18], keeping the notation for the Epileptor network model the same as in previous publications so that they can be easily related. Some of the symbols overlap with the notation for the proposed probabilistic model, but the distinction should be clear from context.

For an *N*-node patient-specific Epileptor network model, the dynamics at each node *i* = 1, 2, …, *N* are given by

$$\begin{array}{c} \begin{array}{c} {\dot{x}}_{1,i}=y_{1,i}-f_{1}\left(x_{1,i},x_{2,i}\right)-z_{i}+I_{1} \end{array} \end{array}$$ (33)

$$\begin{array}{c} \begin{array}{c} {\dot{y}}_{1,i}=\frac{1}{\tau_{1}}\{1-5x_{1,i}^{2}-y_{1,i}\} \end{array} \end{array}$$ (34)

$$\begin{array}{c} \begin{array}{c} {\dot{z}}_{i}=\frac{1}{\tau_{0}}\{4\left(x_{1,i}-x_{0,i}\right)-z_{i}-w\sum_{j=1}^{N}W_{ij}\left[x_{1,j}\left(t-\tau_{ij}\right)-x_{1,i}\left(t\right)\right]\} \end{array} \end{array}$$ (35)

$$\begin{array}{c} \begin{array}{c} {\dot{x}}_{2,i}=-y_{2,i}+x_{2,i}-x_{2,i}^{3}+I_{2}+0.002\;g\left(x_{1,i}\right)-0.3\left(z_{i}-3.5\right)+\xi_{i}\left(t\right) \end{array} \end{array}$$ (36)

$$\begin{array}{c} \begin{array}{c} {\dot{y}}_{2,i}=\frac{1}{\tau_{2}}\left\{-y_{2,i}+f_{2}\left(x_{2,i}\right)\right\}+\eta_{i}\left(t\right), \end{array} \end{array}$$ (37)

where

$$\begin{array}{c} \begin{array}{c} g\left(x_{1,i}\right)=\int_{t_{0}}^{t}e^{-\gamma (t-s)}x_{1,i}\left(s\right)\;ds, \end{array} \end{array}$$ (38)

and

$$\begin{array}{cc} f_{1}\left(x_{1,i},x_{2,i},z_{i}\right) & =\{\begin{array}{cc} x_{1,i}^{3}-3x_{1,i}^{2} & \;\text{if}\;x_{1,i}<0\\ \left(x_{2,i}-0.6{\left(z_{i}-4\right)}^{2}\right)\;x_{1,i} & \;\text{if}\;x_{1,i}\geq 0 \end{array}\\ f_{2}\left(x_{2,i}\right) & =\{\begin{array}{cc} 0 & \;\text{if}\;x_{2,i}<-0.25\\ 6(x_{2,i}+0.25) & \;\text{if}\;x_{2,i}\geq -0.25. \end{array} \end{array}$$

The terms *ξ*_*i*_(*t*) and *η*_*i*_(*t*) correspond to zero mean Gaussian white noise. Also, both *ξ*_*i*_(*t*) and *η*_*i*_(*t*) are independent across nodes. Here, we set the corresponding noise variances to 0.0025. Effectively, we interpret the above as stochastic differential equations in the Itô calculus sense and apply the Euler-Heun method in the stochastic simulations. We set *I*_1_ = 3.1, *I*_2_ = 0.45, *γ* = 0.01, *τ*_0_ = 6667, *τ*_1_ = 1, *τ*_2_ = 10. For agreement with previously published simulations of Epileptor network models, we list the time related variables in time units of 0.02 seconds, e.g. *τ*_1_ = 0.02s.

The coupling weights *W*_*ij*_ ≥ 0 were obtained from patient-specific connectivity matrices derived from white-matter tractography. The terms *τ*_*ij*_ > 0 are the corresponding axonal transmission delays. (For more details, see Structural network connectivity below.) The global connectivity strength is specified by the parameter *w*. We note that, although the connectivity matrix and global coupling consist of nonnegative or strictly positive values, network interactions can result in suppressive or inhibitory effects via a diffusive coupling between nodes instantiated as [*x*_1,*j*_(*t* − *τ*_*ij*_)−*x*_1,*i*_(*t*)] in the above equation for the *z* variable.

Epileptogenic and non-epileptogenic nodes can be instantiated by setting the corresponding node excitability parameter *x*_0,*i*_ to specific distinct values. This excitability parameters control whether a node can go spontaneously into seizure. Its critical value for an isolated node is *x*_0,*b*_ ≈ −2.061. Levels above this value allow for spontaneous seizure transitions. Here, for an epileptogenic zone (EZ) we set *x*_0,*i*_ = {−1.6, −1.8}. For non-epileptogenic nodes in the surround network we considered *x*_0_ ∈ [−2.3, −2.09], with 0.0115 steps.

For a detailed motivation of the Epileptor network equations and their dynamics we refer to [9, 10, 33, 45, 46]. Briefly, three different time scales, reflected in the parameters *τ*_0_ ≫ *τ*_2_ ≫ *τ*_1_, allow the network to capture both the slow and fast oscillations typically observed in epileptic focal seizures. The slowest time scale corresponds to potential ionic, metabolic and homeostatic dynamical processes leading the network to transition into seizures. This slow dynamics is captured by the “permitivity” variable *z*_*i*_(*t*), which effectively works as a bifurcation parameter, leading the network to spontaneously transition in and out of seizures.

### Patient-specific structural network connectivity and interaction delay matrices based on white-matter tractography

Details about (diffusion MRI) white-matter tractography, brain area parcellation, and plots for all of the 5-patient network connectivity and time delay matrices used in this study are provided in Fig A and Table A in S1 Text.

### Fitting the proposed probabilistic model to patient-specific Epileptor network simulation data

The proposed model has in total 6 parameters {*a*, *b*, *c*, *d*, *τ*_*s*_, *h*}, which need to be estimated. To fit these parameters to data from patient-specific Epileptor network models, we simulated Epileptor networks instantiated on 5 different patient-specific networks. For each network, we considered two different cases of EZ node location. For each network, we also considered two different EZ excitability levels set at *x*_0,*ez*_ = {−1.6, −1.8}. In total we had 20 different networks. In addition, we varied the control parameters (*w*, *x*_0_), i.e. the excitability and global connectivity strength, respectively, on a grid space. For each point on these grid spaces and for each network, we simulated 20 stochastic realizations and computed the corresponding phase-diagrams as shown in Fig 2.

As stated earlier, we emphasize that we fitted just a single model to all of the simulated patient-specific Epileptor networks, choices of EZ node location, and variations in excitability and global connectivity strength. First, to fit the parameters {*τ*_*s*_, *c*, *d*}, which specify the duration of seizures and their variability, we considered the seizure duration in the EZ node *D*_*ez*_ in the no-spread phase region of the (*w*, *E*) space. In that region, the seizure starts in the EZ node but does not spread (green area in Fig 2). Furthermore, in this region of the phase diagram, the input to the EZ node is constant over time

$$\begin{array}{c} I_{ez}\left(t\right)=I_{ez}=wE\sum_{i}W_{ez,i}. \end{array}$$

Thus, we can simply calculate the probability distribution function of *D*_*ez*_ during this phase of the network using Eqs 5, 10, 11 and 12 by considering *y* = *t* − *t*_*so*,*ez*_ as the time elapsed since the seizure initiation, the relation between the duration of seizure and rate of termination is

$$\begin{array}{c} p\left(D_{ez}\right)=g\left(D_{ez},{\tilde{\tau }}_{s,ez},q_{s,ez}\right)\text{exp}(-\int_{0}^{D_{ez}}g\left(y,{\tilde{\tau }}_{s,ez},q_{s,ez}\right)dy). \end{array}$$

Because in this parameter range ${\tilde{\tau }}_{s,ez}$ and *q*_*s*,*ez*_ are independent of time, direct calculation leads to a uniform distribution of *D*_*ez*_ in the range $\left[{\tilde{\tau }}_{s,ez}-q_{s,ez},{\tilde{\tau }}_{s,ez}+q_{s,ez}\right)$ with mean ${\tilde{\tau }}_{s,ez}$ and standard deviation

$$\begin{array}{c} \sigma_{D_{ez}}=\frac{q_{s,ez}}{\sqrt{3}}. \end{array}$$

Next, we examined the behavior of *D*_*ez*_ as a function of the input to the EZ node (Fig O panel A in S1 Text). Furthermore, the expected value conditioned on *I*_*ez*_ and standard deviation of *D*_*ez*_ are both in agreement with the functional form of Eqs 11 and 12 (Fig O panels B and C in S1 Text). By fitting the functions directly to the data, we estimated *τ*_*s*_ = 32.22*s*, *c* = 1.3 and *d* = 0.05.

Having determined *τ*_*s*_, to fit the parameters *a*, *b* we extracted the phase transition boundary (the boundary between green and yellow in Fig 2) from all of the phase diagrams constructed for all patient-specific networks and corresponding EZ nodes from the Epileptor simulations. For each phase diagram, the data were constructed as the set of pairs {(*E*_*k*,*r*_, *w*_*k*,*r*_)}, where the indexes *k* and *r* indicate different points on the given transition boundary and different stochastic realizations, respectively. We used least-squares to fit parameters *a* and *b*. Specifically, we used Eq 17 in the following quadratic cost function to be minimized with respect to *a* and *b*

$$\begin{array}{c} L\left(a,b\right)=\sum_{P}\sum_{EZ}\sum_{k}\sum_{r}{\left(E_{k,r}+\frac{w_{k,r}aW_{i,ez}{\tilde{\tau }}_{s}}{\tau_{s}\left[1+w_{k,r}b\sum_{j\notin EZ}W_{ij}\right]}\right)}^{2}, \end{array}$$

leading to *a* = 0.46 and *b* = 0.0021. We note that the index *i* in the above cost function specifies the most susceptible node, which is identified by finding the node with maximum input from the EZ node. In the above equation the first and second summations are with respect to patients and EZ nodes, respectively.

In order to fit the remaining parameter *h*, we extracted the “inhibitory” boundary (i.e. boundary of the blue region in Fig 2) from each of phase diagrams obtained from the Epileptor network simulations. For each case, the data were constructed as the set of pairs of {(*w*_*j*,*r*_, *E*_*j*,*r*_)} where the indexes *j* and *r* indicate different points on the phase boundary and different stochastic realizations, respectively. Using Eq 16 and setting

$$\begin{array}{c} E_{ez}=\left(x_{0,ez}-x_{0,b}\right)h, \end{array}$$

we minimized the following quadratic cost function

$$\begin{array}{c} L\left(h\right)=\sum_{P}\sum_{EZ}\sum_{j}\sum_{r}{\left(E_{j,r}+\frac{(x_{0,ez}-x_{0,b})h}{w_{j,r}b\sum_{i}W_{ez,i}}\right)}^{2}, \end{array}$$

resulting in *h* = 0.01.

### Numerical simulations: Temporal Gillespie algorithm

To simulate in continuous-time the exact dynamics of the probabilistic model we used an algorithm based on an extension of the time-rescaling theorem [26, 27], also known as the temporal Gillespie algorithm [28]. Consider first just a single-node (i.e. *N* = 1) with a corresponding set **Ω** of possible state transitions including seizure initiation, termination (transition to refractory or postictal) and recovery. Any realization of the process consists of a sequence of transition times

$$\begin{array}{c} \{t_{1},t_{2},t_{3},\ldots \}, \end{array}$$

which can be represented as a realization from a stochastic point process with conditional intensity

$$\begin{array}{c} \Lambda \left(t|H\left(t\right)\right)=\sum_{\omega \in \Omega }\lambda_{\omega }\left(t|H\left(t\right)\right), \end{array}$$

where H(t) is the history of the process, and $\lambda_{\omega }\left(t|H\left(t\right)\right)$ is the conditional intensity for each of the possible *ω* ∈ *Ω* transition types. The $\lambda_{\omega }\left(t|H\left(t\right)\right)$ are specified by the corresponding rate functions *f* and *g*, i.e. Eqs 6 and 10, with appropriate input arguments.

By the time-rescaling theorem, the rescaled (waiting) time intervals between transitions

$$\begin{array}{c} \upsilon_{k}=\int_{t_{k}}^{t_{k+1}}\Lambda \left(t|H\left(t\right)\right)\;dt \end{array}$$ (39)

are independent and exponentially distributed with mean 1, i.e. the rescaled-time point process corresponds to a homogeneous Poisson process with unit rate. Thus, to simulate the original process, one can simply start by sampling a sequence of waiting time intervals {*υ*_*k*_} from this unit mean exponential distribution, and for each one of them, use Eq 39 to solve for the transition times. Concretely, given a transition time *t*_1_ and a new sampled waiting time *υ*_1_, one solves Eq 39 for the next transition time *t*_2_. Next, the type of the transition itself, indicated by *ω*, is sampled according to the probabilities

$$\begin{array}{c} P_{\omega }=\frac{\lambda_{\omega }\left(t_{2}|H\left(t_{2}\right)\right)}{\Lambda (t_{2}|H\left(t_{2}\right))}. \end{array}$$

The simulation continues from one transition to the other by repeating the above steps.

The above can be easily extended to networks, by setting

$$\begin{array}{c} \Lambda \left(t|H\left(t\right)\right)=\sum_{\omega \in \Omega }\sum_{j=1}^{N}\lambda_{j,\omega }\left(t|H\left(t\right)\right), \end{array}$$

where λ_*j*,*ω*_ corresponds to the intensity at node *j*, and using again Eq 39 to solve for the transition times. Finally, both the node and the type of the transition are sampled with probabilities

$$\begin{array}{c} P_{j,\omega }=\frac{\lambda_{j,\omega }\left(t_{k}|H\left(t_{k}\right)\right)}{\Lambda (t_{k}|H\left(t_{k}\right))}, \end{array}$$

where *t*_*k*_ is the currently sampled transition time under consideration. The simulation proceeds following the above steps.

We emphasize that the seizure always starts in the EZ nodes. Furthermore, when applying the algorithm to ER random networks (as opposed the patient-specific networks), we enforce a seizure to start simultaneously in all of the *N*_*ez*_ = *ϵN* EZ nodes.

### Calculation of the spread to no-spread boundary in the mean-field approximations

To evaluate the boundary between seizure spread and no-spread, we start with the case where the EZ nodes are in the seizure state. We want to find the points in the parameter space (*E*, *w*) at which the probability of spread is zero. We refer to the boundary of this region as the no-spread to spread boundary. Assuming that the EZ nodes are in the seizure state, following the same steps as in deriving Eq 17, and considering the node with largest *z*_*i*_(*t*), we can write the equation for this boundary as

$$\begin{array}{c} E=\frac{-wa\max_{t}\left\{\sum_{j\in \text{EZ}}W_{ij}u_{j}\left(t-\tau_{ij}\right)\right\}}{\left[1+wb\sum_{j\notin \text{EZ}}W_{ij}\right]}. \end{array}$$ (40)

While the above equation is written for the node with largest *z*_*i*_(*t*), in the mean-field approximation all the surrounding nodes receive equal input from the EZ nodes. Therefore we are not concerned with the maximization with respect to *i*. To derive the mean-field approximation of the boundary between no-spread and spread phases, we replace *W*_*ij*_ with its average value $\bar{W}$ and replace the interaction delays *τ*_*ij*_ with their average value $\bar{\tau }$. Additionally, since the the summation in the denominator of the above equation does not include the EZ nodes, it can be evaluated as $\sum_{j\notin \text{EZ}}W_{ij}=\bar{W}\left(N-N_{ez}\right)$. Thus

$$\begin{array}{c} E=\frac{-w\bar{W}aN_{ez}\max_{t}\left\{\sum_{j\in \text{EZ}}u_{j}\left(t-\bar{\tau }\right)/N_{ez}\right\}}{1+wb\bar{W}\left(N-N_{ez}\right)}. \end{array}$$ (41)

Next, to get the final form of this boundary we need to find the maximum of

$$\begin{array}{c} U\left(t\right)=\sum_{j\in \text{EZ}}u_{j}\left(t\right)/N_{ez}. \end{array}$$ (42)

with respect to time. Note that as the translation in time with $\bar{\tau }$ does not change the maximum value of this function, for convenience we do not consider it in the following calculations. In our setup, we consider the case that all the EZ nodes go to seizure simultaneously at time zero. (This reflects the observation that the time scale for seizure spread within the EZ region is much faster than the time scale for seizure spread in the large-scale network.) In addition, in the no-spread phase, the duration of seizures in the EZ nodes is a random variable uniformly distributed in the range $\left({\tilde{\tau }}_{s,ez}-q_{s,ez},{\tilde{\tau }}_{s,ez}+q_{s,ez}\right)$. Based on the above, we can evaluate an approximation of *U*(*t*) that is exact in the *N*_*ez*_ → ∞ limit. To achieve that, we divide the time axis into 4 intervals, and for each interval we calculate the function *U*(*t*). See Fig P in S1 Text for visualization and a summary of the following results. The intervals and related calculations are:

1. Time interval $0\leq t<{\tilde{\tau }}_{s,ez}-q_{s,ez}$: All the EZ nodes are in the seizure state and all have the same *u*_*i*_(*t*) value. Therefore,
  $$\begin{array}{c} U\left(t\right)=\frac{t-t_{\text{so},ez}}{\tau_{s}}=\frac{t}{\tau_{s}} \end{array}$$
2. Time interval ${\tilde{\tau }}_{s,ez}-q_{s,ez}\leq t<{\tilde{\tau }}_{s,ez}+q_{s,ez}$: The uniformly-distributed (for large *N*_*ez*_) seizure termination times for EZ nodes can be approximated as homogeneous termination times, which are proportional to the time passed since ${\tilde{\tau }}_{s,ez}-q_{s,ez}$. In other words if $\phi \left(t\right)=t-\left({\tilde{\tau }}_{s,ez}-q_{s,ez}\right)$ and Φ = 2*q*_*s*,*ez*_, then the ratio of EZ nodes that have undergone seizure termination up to time *t* is equal to $\frac{\phi (t)}{\Phi }$, and the ratio of nodes that are still in the seizure phase is $1-\frac{\phi (t)}{\Phi }$. In this time interval, the contribution of the nodes that are still in the seizure state to *U*(*t*) is $\frac{t}{\tau_{s}}\left(1-\frac{\phi (t)}{\Phi }\right)$. Using the fact that for the rest of the nodes the {*u*_*i*_(*t*)} are in their linear decreasing phase, and also the uniform termination times, the average contribution of these nodes is equal to $\frac{\phi (t)}{\Phi }\left({\tilde{\tau }}_{s,ez}-q_{s,ez}\right)$. Therefore,
  $$\begin{array}{c} U\left(t\right)=\frac{1}{\tau_{s}}\left[\left(1-\frac{\phi (t)}{\Phi }\right)t+\frac{\phi (t)}{\Phi }\left({\tilde{\tau }}_{s,ez}-q_{s,ez}\right)\right]. \end{array}$$
  Since for $t>{\tilde{\tau }}_{s,ez}+q_{s,ez}$ all $\left\{u_{i}\left(t\right)|i\in \text{EZ}\right\}$ are in their decreasing phase, the maximum point of *U*(*t*) is expected to be in the above intervals. In addition, the maximum point is not in the first interval because $U\left(t\right)<U\left({\tilde{\tau }}_{s,ez}-q_{s,ez}\right)$ for any $t<{\tilde{\tau }}_{s,ez}-q_{s,ez}$. Therefore, the maximum point must be in the second interval. By calculating the root of the time derivative of *U*(*t*), which is a quadratic function with a unique extremum point in the interval, we can show that the maximum point happens at $t={\tilde{\tau }}_{s,ez}$. Thus
  $$\begin{array}{c} U_{max}=\frac{{\tilde{\tau }}_{s,ez}-q_{s,ez}/2}{\tau_{s}}. \end{array}$$
  For completeness, to show the calculation of *U*(*t*) at all times, we also evaluate it here in the following two intervals.
3. Time interval ${\tilde{\tau }}_{s,ez}+q_{s,ez}\leq t<2\left({\tilde{\tau }}_{s,ez}-q_{s,ez}\right)$: The {*u*_*i*_(*t*)} for all the nodes are in their decreasing phase, and due to the uniform distribution of the termination times, their average is equal to
  $$\begin{array}{c} U\left(t\right)=\frac{2{\tilde{\tau }}_{s,ez}-t}{\tau_{s}} \end{array}$$
4. Time interval $2\left({\tilde{\tau }}_{s,ez}-q_{s,ez}\right)\leq t<2\left({\tilde{\tau }}_{s,ez}+q_{s,ez}\right)$: The {*u*_*i*_(*t*)} of some of the nodes are in their linearly decreasing phase and *u*_*i*_(*t*) = 0 for the rest of the nodes. The ratio of the nodes for which *u*_*i*_(*t*) = 0, during times in this interval, is proportional to the time that has passed since $2({\tilde{\tau }}_{s,ez}-q_{s,ez})$, i.e. $\frac{t-2({\tilde{\tau }}_{s,ez}-q_{s,ez})}{2\Delta }$. The ratio of the nodes that are in the decreasing phase is $1-\frac{t-2({\tilde{\tau }}_{s,ez}-q_{s,ez})}{2\Delta }$. Therefore, the average *u*_*i*_(*t*) of these nodes is equal to
  $$\begin{array}{c} U\left(t\right)=\left(1-\frac{t-2({\tilde{\tau }}_{s,ez}-q_{s,ez})}{2\Phi }\right)\frac{2({\tilde{\tau }}_{s,ez}+q_{s,ez})-t}{2\tau_{s}}. \end{array}$$

Outside of the union of these four intervals, *u*_*i*_(*t*) = 0 for all the EZ nodes and obviously *U*(*t*) = 0.

Having derived the maximum value of *U*(*t*) we continue the calculation of the boundary of spread to no-spread. By plugging $U_{\text{max}}=\max_{t}\left\{\sum_{j\in \text{EZ}}u_{j}\left(t\right)/N_{ez}\right\}=\left({\tilde{\tau }}_{s,ez}-q_{s,ez}/2\right)/\tau_{s}$ into Eq 41, we get the boundary of no-spread to spread as

$$\begin{array}{c} E=\frac{-w\bar{W}aN_{ez}\left({\tilde{\tau }}_{s,ez}-q_{s,ez}/2\right)}{\tau_{s}\left[1+wb\left(N-N_{ez}\right)\bar{W}\right]}. \end{array}$$ (43)

Based on the mean-field approximation for large random networks with connection probability *p*, with a fraction of EZ nodes *ϵ* = *N*_*ez*_/*N* and connectivity weights sampled from a distribution with average *μ*_0_/*N*, we get $\bar{W}=p\mu_{0}/N$. Plugging $\bar{W}$ into the above equation, we can rewrite the boundary equation as

$$\begin{array}{c} E=\frac{-wap\mu_{0}\epsilon \left({\tilde{\tau }}_{s,ez}-q_{s,ez}/2\right)}{\tau_{s}\left(1+wbp\mu_{0}\left(1-\epsilon \right)\right)}. \end{array}$$ (44)

### Finite-size correction for mean-field derived phase diagrams

We present the detailed derivation of the finite-size correction Eq 25 for the mean-field prediction of phase diagrams for the proposed model. Having a fraction *ϵ* of all the nodes in a random network, with connection probability *p*, as EZ nodes, the total number of edges from all the EZ nodes to a node in the surrounding is distributed according to a binomial distribution with mean

$$\begin{array}{c} {\bar{K}}_{\text{EZ}}=\epsilon pN \end{array}$$

and standard deviation

$$\begin{array}{c} \sigma_{K_{\text{EZ}}}=\sqrt{pN\;\epsilon (1-\epsilon )}. \end{array}$$

Multiplying the total number of edges by the average connection weight per edge (*μ*_0_/*N*), we get the average and standard deviation of the input from EZ nodes to a random node in the network as

$$\begin{array}{c} {\bar{W}}_{\text{EZ}}=wp\epsilon \mu_{0} \end{array}$$

and

$$\begin{array}{c} \sigma_{W_{\text{EZ}}}=w\mu_{0}\sqrt{p\epsilon (1-\epsilon )/N}, \end{array}$$

respectively.

For finite *N* there is variability in the inputs from the EZ node to surrounding nodes, and there is a probability of having non-EZ nodes that receive inputs larger than ${\bar{W}}_{\text{EZ}}$. A finite-size correction to Eq 23 can thus be obtained by considering such nodes as the most susceptible nodes with inputs up to *n* standard deviations larger than the mean input, leading to

$$\begin{array}{cc} w\sum_{j\in \text{EZ}}W_{ij} & \approx wp\epsilon \mu_{0}+nw\mu_{0}\sqrt{p\epsilon (1-\epsilon )/N}\\ & =wp\epsilon \mu_{0}\left(1+n\sqrt{\frac{\left(1-\epsilon \right)}{\epsilon pN}}\right). \end{array}$$ (45)

Therefore the finite-size correction is obtained by multiplying the right hand side of Eq 24 by the correction factor

$$\begin{array}{c} \nu =1+n\sqrt{\frac{\left(1-\epsilon \right)}{\epsilon pN}}. \end{array}$$

### Mean-field dynamics

Mean-field analysis is based on the assumption that the effective input to a node from others at a given time is the same for all the nodes in the system. In other words, if we define

$$\begin{array}{c} {\bar{W}}_{i}=\frac{1}{N}\sum_{j=1}^{N}W_{ij}, \end{array}$$ (46)

then under the mean-field assumption ${\bar{W}}_{i}={\bar{W}}_{j}$ for all pairs of the nodes. Therefore, we drop the index of this mean value and use $\bar{W}$. Now, we can write the mean-field expression for *z*_*i*_(*t*) as

$$\begin{array}{c} z_{i}\left(t\right)=z\left(t\right)=\bar{W}wa\sum_{j=1}^{N}u_{j}\left(t-\bar{\tau }\right)+\bar{W}wbE\sum_{j=1\notin \text{EZ}}^{N}v_{j}\left(t-\bar{\tau }\right), \end{array}$$ (47)

where $\bar{\tau }$ is the average interaction delay over all pairs of nodes defined as

$$\begin{array}{c} \bar{\tau }=\frac{\sum_{i,j}\tau_{ij}}{\sum_{i,j}A_{ij}}, \end{array}$$ (48)

where *A*_*ij*_ are binary elements of the adjacency matrix (i.e. *A*_*ij*_ = 1 if there is a connection form *j* to *i*, and zero otherwise). Using the above equation and Eqs 3–5, we derive the mean-field equations for evolution of the number of nodes in each of the three possible states of the model.

To do that, we make a discrete-time approximation and study the evolution of the system over small time intervals of size $\Delta =\bar{\tau }/m_{\tau }$, where *m*_*τ*_ is the number of time steps in the delay time interval. We define the number of non-EZ nodes that transition to the seizure state at each time bin *i* as *M*_*i*_, the number of non-EZ nodes that enter the seizure state at time bin *i* and exit this state at time bin *j* (seizure ending) as *n*_*ij*_, and the number of non-EZ nodes that exit the seizure state in time bin *i* and recover from refractory state in time bin *j* as *r*_*ij*_. Similarly, we define the same variables for the EZ nodes as $M_{i}^{ez}$, $n_{ij}^{ez}$ and $r_{ij}^{ez}$.

Next, using Eqs 8 and 9, we can write *z*_*m*_ = *z*(*m*Δ) in terms of the above variables as

$$\begin{array}{cc} z_{m} & =\frac{\bar{W}wa\Delta }{\tau_{s}}\sum_{i=-\infty }^{m-m_{\tau }}\{\left(m-i\right)\left[M_{i}+M_{i}^{ez}-\sum_{j=i}^{m}\left(n_{ij}+n_{ij}^{ez}\right)\right]\\ & +\sum_{j=i}^{m}H\left(2j-i-m\right)\left(n_{ij}+n_{ij}^{ez}\right)\}+\bar{W}wbEN_{s,m}, \end{array}$$ (49)

where *N*_*s*,*m*_ = *N*_*s*_(*m*Δ) is the number of non-EZ nodes in the susceptible state in the *m*^th^ time bin. The term

$$\begin{array}{c} M_{i}+M_{i}^{ez}-\sum_{j=i}^{m}\left(n_{ij}+n_{ij}^{ez}\right) \end{array}$$

in Eq 49 gives the number of nodes that have entered the seizure state at the time bin *i* and are still in this state at time bin *m*. It accounts for the linear increase of the output from these nodes to the neighbor nodes before seizure termination. On the other hand, the term

$$\begin{array}{c} \sum_{j=i}^{m}H\left(2j-i-m\right)\left(n_{ij}+n_{ij}^{ez}\right) \end{array}$$

accounts for the linear decrease in the accumulated input after seizure termination (see Eq 8). The function

$$\begin{array}{c} H\left(x\right)=\{\begin{array}{cc} x & x\geq 0\\ 0 & x<0 \end{array} \end{array}$$ (50)

guarantees that this linear decrease stops at zero.

By denoting the history of the system up to and including time bin *m* as

$$\begin{array}{c} H_{m}=\left\{M_{i},n_{ij},r_{ij},M_{i}^{ez},n_{ij}^{ez},r_{ij}^{ez}|i\leq m\;\text{and}\;j\leq i\right\}, \end{array}$$

we can write the probability of having *M*_*m*+1_ non-EZ nodes transitioning to seizure in time bin *m* + 1 in terms of a binomial distribution

P(Mm+1|Hm)=(Ns,mMm+1)[f(zm+E)Δ]Mm+1×[1-f(zm+E)Δ]Ns,m-Mm+1. (51)

We write the above probability function based on the fact that there are *N*_*s*,*m*_ susceptible nodes at time bin *m*, out of which *M*_*m*+1_ nodes are chosen to transition to the seizure state at time bin *m* + 1 where each transition takes place with probability *f*(*z*_*m*_ + *E*)Δ.

Similarly, we can write the probability of having *n*_*i*,*m*+1_ non-EZ nodes, from the *M*_*i*_ nodes that have gone to the seizure state in the time bin *i* and are still in the seizure state, transitioning to the refractory state at time bin *m* + 1 as

P(ni,m+1|Hm)=(Mi-∑j=imnijni,m+1)[g(Δ(m-i),τ˜s,m,qs,m)Δ]ni,m+1×[1-g(Δ(m-i),τ˜s,m,qs,m)Δ]Mi-∑j=im+1nij, (52)

where

$$\begin{array}{c} {\tilde{\tau }}_{s,m}=\tau_{s}/\left(1-cEw\bar{W}N_{s,m}\right) \end{array}$$

is the mean-field approximation of Eq 11 at time bin *m* and $q_{s,m}=d{\tilde{\tau }}_{s,m}$ is related to the variability of seizure termination times. In the above equation $\bar{W}$ is the average interaction weight.

Finally, the probability of having *r*_*j*,*m*+1_ non-EZ nodes, out of all the nodes that have transitioned to the refractory state in time bin *j* and have not recovered yet, transitioning from the refractory to the susceptible state at time bin *m* + 1 can be written as

P(rj,m+1|Hm)=(∑i=-∞jnij-∑k=jmrjkrj,m+1)[g([m-j]Δ,τ˜r,m,qr,m)Δ]rj,m+1×[1-g([m-j]Δ,τ˜r,m,qr,m)Δ]∑i=-∞jnij-∑k=jm+1rjk. (53)

where ${\tilde{\tau }}_{r,m}=\tau_{r}$ is the refractory time scale and *q*_*r*,*m*_ = *q*_*r*_ specifies the respective variability in the refractory time.

At a time bin *m* we can evaluate the number of nodes in seizure *N*_*e*,*m*_, susceptible *N*_*s*,*m*_ and refractory states *N*_*r*,*m*_ as

$$\begin{array}{c} N_{e,m}=\sum_{i=-\infty }^{m}(M_{i}-\sum_{j=i}^{m}n_{ij}) \end{array}$$ (54)

$$\begin{array}{c} N_{r,m}=\sum_{j=-\infty }^{m}(\sum_{i=-\infty }^{j}n_{ij}-\sum_{k=j}^{m}r_{jk}) \end{array}$$ (55)

and

$$\begin{array}{c} N_{s,m}=N-N_{ez}-N_{r,m}-N_{e,m}, \end{array}$$ (56)

respectively.

Eqs 51–53 can be similarly written for the EZ nodes represented by the variables $M_{i}^{ez}$, $n_{ij}^{ez}$ and $r_{ij}^{ez}$ defined above.

### Sparse-seizure correction to mean-field dynamics

We developed a correction to Eqs 26 and 49 that is valid in the regime of spreading dynamics where there is a small number of output edges from seizing nodes to susceptible nodes compared with the total number of susceptible nodes in the network. Examples of this regime are the early time of spread dynamics on networks with small number of EZ nodes, e.g. patient-specific networks examined here, and sparse random networks with small average degree.

For a random network with connectivity probability *p*, the spread dynamics at time bin *m* is determined by the number of seizing nodes *N*_*e*,*m*_ and the number of susceptible nodes *N*_*s*,*m*_. The expected number of output links projecting from the *N*_*e*,*m*_ active nodes to *N*_*s*,*m*_ susceptible nodes can be written as *K*_*se*,*m*_ = *pN*_*e*,*m*_*N*_*s*,*m*_, where the subscript _*se*_ indicates the links or edges from excited to susceptible nodes. If *N*_*s*,*m*_ > *K*_*se*,*m*_, then the output from the active nodes does not evenly distribute among all the *N*_*s*_ nodes; instead it can at most affect *K*_*se*,*m*_ of the *N*_*s*,*m*_ susceptible nodes. Therefore, we can make a correction to Eq 26 where instead of $\left(\begin{array}{c} N_{s,m}\\ M_{m+1} \end{array}\right)$ we use $\left(\begin{array}{c} K_{se,m}\\ M_{m+1} \end{array}\right)$.

In addition, we can also make a correction to Eq 49. Based on the above argument, the excitatory output from the *N*_*e*,*m*_ nodes can at most excite *K*_*se*,*M*_ nodes. As a consequence, we can rescale the excitatory part of *z*_*m*_ in Eq 49, which is evaluated to distribute among all susceptible nodes by a factor of

$$\begin{array}{c} \frac{N_{s,m}}{K_{se,M}}. \end{array}$$

Obviously, the above corrections are valid only if *N*_*s*,*m*_ > *K*_*se*,*m*_. Otherwise, the dynamics follows the originally derived mean-field dynamics. We used mean-field simulations with the above corrections to approximate the spreading dynamics in patient-specific networks as shown in Fig 4C.

### Simulation of mean-field dynamics

To simulate the mean-field dynamics, one can start from any initial condition and sample the binomial distributions in Eqs 26–28 together with the corresponding equations for the EZ nodes (overall 6 equations), over small time steps of size $\Delta =\bar{\tau }/m_{\tau }$. Here, we used computer simulations to study the spread dynamics conditioned on seizure initiation at the EZ nodes at time *t* = 0. In addition, because of the separation of time-scales in the model, i.e. ${\tilde{\tau }}_{r,i}\gg {\tilde{\tau }}_{s,i}$, we considered different seizure spread events, separated by a long refractory period, as independent. Therefore, each realization starts with seizure initiation at the EZ nodes if EZ nodes are not inhibited (i.e. *f*(*z*_0_ + *E*_*ez*_)>0), and the dynamics goes on until there is no seizure in the system.

### Behavior of *ψ*_−_ (apparent exponents)

For the behavior of *ψ*_−_ (Eqs 29 and 31), the estimated exponents ${\hat{\beta }}^{\prime }$ and ${\hat{\delta }}^{\prime }$ were negative. Negative values lead to a singularity in the function for *ψ*. Obviously, this type of singularity is not allowed. We present below a consistent formulation of the behavior of the order parameter spread size below the critical point *w* < *w*_*c*_ where negative exponents were estimated. We follow the concept of apparent exponents as described in [32] in the context of estimating power-law probability density functions.

Letting *x* = *w*_*c*_ − *w*, then we must have *ψ*(*x* = 0) = *ψ*_*c*_ which in our model corresponds to a positive finite value. In addition, the response function

$$\begin{array}{c} \chi_{w}=\frac{\partial \psi }{\partial w}=-\frac{\partial \psi }{\partial x} \end{array}$$

is expected to diverge at the critical point. Therefore, it follows that

$$\begin{array}{cc} \psi (x=0) & =\psi_{c} \end{array}$$ (57)

$$\begin{array}{cc} \underset{x\to 0^{+}}{\text{lim}}\chi_{w} & =+\infty . \end{array}$$ (58)

Based on the above conditions, consider next the following functional form for the order parameter:

$$\begin{array}{c} \psi \left(x\right)=\psi_{c}[1-x^{{\hat{\beta }}^{\prime }}G({\left(\frac{x}{x_{c}}\right)}^{\theta })]. \end{array}$$ (59)

The function *G* must be in agreement with conditions 57 and 58. It is necessary that

$$\begin{array}{cc} & G(0)=0\\ & G^{\prime }\left(0\right)=A, \end{array}$$ (60)

for finite positive *A*. In addition, it is necessary that $\theta +{\hat{\beta }}^{\prime }>0$, which guarantees condition in Equation Eq 57. Using Eq 59, it is straightforward to show that the actual exponent governing the behavior of *ψ* near the critical point *x* ≳ 0 is $\beta^{\prime }=\theta +{\hat{\beta }}^{\prime }$.

$$\begin{array}{c} \psi_{-}∼x^{\beta^{\prime }}=x^{\theta +{\hat{\beta }}^{\prime }}. \end{array}$$ (62)

The parameter *x*_*c*_ in the above is a positive number that specifies the scale of *x* such that for *x* > *x*_*c*_, the function *G* approaches a constant value, thus leading to the observation of the apparent exponent ${\hat{\beta }}^{\prime }$. For small *x* < *x*_*c*_ near *x* = 0, the function *G* scales as *x*^*θ*^, thus adding the value of *θ* to the apparent exponent ${\hat{\beta }}^{\prime }$.

From Eq 59 we can also calculate the response function whose behavior near the critical point *x* ≳ 0 is

$$\begin{array}{c} \chi_{w-}∼x^{\theta +\hat{\beta }-1}, \end{array}$$ (63)

which diverges at the critical point if $\theta +\hat{\beta }<1$. Therefore, the intersection of conditions in Eqs 57 and 58 dictates

$$\begin{array}{c} 0<\theta +\hat{\beta }<1. \end{array}$$ (64)

The above argumentation can be similarly applied to the ${\hat{\delta }}^{\prime }$ exponent.

## Supporting information

S1 Text Supplementary text, figures and table describing data and additional results. (PDF)

## References

[1] Thurman DJ, Beghi E, Begley CE, Berg AT, Buchhalter JR, Ding D, et al. Standards for epidemiologic studies and surveillance of epilepsy. Epilepsia. 2011;52(s7):2–26. doi: 10.1111/j.1528-1167.2011.03121.x 21899536

[2] England MJ, Liverman CT, Schultz AM, Strawbridge LM. Epilepsy across the spectrum: Promoting health and understanding.: A summary of the Institute of Medicine report. Epilepsy & Behavior. 2012;25(2):266–276. doi: 10.1016/j.yebeh.2012.06.016 23041175 PMC3548323

[3] Engel Jr J. The current place of epilepsy surgery. Current opinion in neurology. 2018;31(2):192. doi: 10.1097/WCO.0000000000000528 29278548 PMC6009838

[4] Culler IV GW, Jobst BC. Surgical Treatments for epilepsy. CONTINUUM: Lifelong Learning in Neurology. 2022;28(2):536–558. 35393969 10.1212/CON.0000000000001106

[5] Touma L, Dansereau B, Chan AY, Jetté N, Kwon CS, Braun KP, et al. Neurostimulation in people with drug-resistant epilepsy: Systematic review and meta-analysis from the ILAE Surgical Therapies Commission. Epilepsia. 2022;. 10.1111/epi.17243 35352349

[6] Fisher R, Salanova V, Witt T, Worth R, Henry T, Gross R, et al. Electrical stimulation of the anterior nucleus of thalamus for treatment of refractory epilepsy. Epilepsia. 2010;51(5):899–908. doi: 10.1111/j.1528-1167.2010.02536.x 20331461

[7] Skarpaas TL, Jarosiewicz B, Morrell MJ. Brain-responsive neurostimulation for epilepsy (RNS® System). Epilepsy research. 2019;153:68–70. doi: 10.1016/j.eplepsyres.2019.02.003 30850259

[8] Fisher B, DesMarteau JA, Koontz EH, Wilks SJ, Melamed SE. Responsive vagus nerve stimulation for drug resistant epilepsy: a review of new features and practical guidance for advanced practice providers. Frontiers in neurology. 2021; p. 1863. doi: 10.3389/fneur.2020.610379 33584511 PMC7874068

[9] Jirsa VK, Stacey WC, Quilichini PP, Ivanov AI, Bernard C. On the nature of seizure dynamics. Brain. 2014;137(8):2210–2230. doi: 10.1093/brain/awu133 24919973 PMC4107736

[10] Proix T, Bartolomei F, Chauvel P, Bernard C, Jirsa VK. Permittivity coupling across brain regions determines seizure recruitment in partial epilepsy. Journal of Neuroscience. 2014;34(45):15009–15021. doi: 10.1523/JNEUROSCI.1570-14.2014 25378166 PMC6608363

[11] Proix T, Jirsa VK, Bartolomei F, Guye M, Truccolo W. Predicting the spatiotemporal diversity of seizure propagation and termination in human focal epilepsy. Nature communications. 2018;9(1):1–15. doi: 10.1038/s41467-018-02973-y 29540685 PMC5852068

[12] Sip V, Guye M, Bartolomei F, Jirsa V. Computational modeling of seizure spread on a cortical surface. Journal of Computational Neuroscience. 2022;50(1):17–31. doi: 10.1007/s10827-021-00802-8 34686937 PMC8818012

[13] Jirsa VK, Proix T, Perdikis D, Woodman MM, Wang H, Gonzalez-Martinez J, et al. The virtual epileptic patient: individualized whole-brain models of epilepsy spread. Neuroimage. 2017;145:377–388. doi: 10.1016/j.neuroimage.2016.04.049 27477535

[14] Proix T, Bartolomei F, Guye M, Jirsa VK. Individual brain structure and modelling predict seizure propagation. Brain. 2017;140(3):641–654. doi: 10.1093/brain/awx004 28364550 PMC5837328

[15] An S, Bartolomei F, Guye M, Jirsa V. Optimization of surgical intervention outside the epileptogenic zone in the Virtual Epileptic Patient (VEP). PLoS computational biology. 2019;15(6):e1007051. doi: 10.1371/journal.pcbi.1007051 31242177 PMC6594587

[16] Olmi S, Petkoski S, Guye M, Bartolomei F, Jirsa V. Controlling seizure propagation in large-scale brain networks. PLoS computational biology. 2019;15(2):e1006805. doi: 10.1371/journal.pcbi.1006805 30802239 PMC6405161

[17] Hashemi M, Vattikonda A, Sip V, Guye M, Bartolomei F, Woodman M, et al. The Bayesian Virtual Epileptic Patient: A probabilistic framework designed to infer the spatial map of epileptogenicity in a personalized large-scale brain model of epilepsy spread. NeuroImage. 2020;. doi: 10.1016/j.neuroimage.2020.116839 32387625

[18] Moosavi SA, Jirsa VK, Truccolo W. Critical dynamics in the spread of focal epileptic seizures: Network connectivity, neural excitability and phase transitions. Plos one. 2022;17(8):e0272902. doi: 10.1371/journal.pone.0272902 35998146 PMC9397939

[19] Christensen K, Moloney NR. Complexity and criticality. vol. 1. World Scientific Publishing Company; 2005.

[20] Livi R, Politi P. Nonequilibrium statistical physics: a modern perspective. Cambridge University Press; 2017.

[21] Bak P, Tang C, Wiesenfeld K. Self-organized criticality: An explanation of the 1/f noise. Physical review letters. 1987;59(4):381. doi: 10.1103/PhysRevLett.59.381 10035754

[22] Zapperi S, Lauritsen KB, Stanley HE. Self-organized branching processes: mean-field theory for avalanches. Physical review letters. 1995;75(22):4071. doi: 10.1103/PhysRevLett.75.4071 10059807

[23] Dickman R, Vespignani A, Zapperi S. Self-organized criticality as an absorbing-state phase transition. Physical Review E. 1998;57(5):5095. doi: 10.1103/PhysRevE.57.5095

[24] Hethcote HW. The mathematics of infectious diseases. SIAM review. 2000;42(4):599–653. doi: 10.1137/S0036144500371907

[25] Sadurní E, Luna-Acosta G. Exactly solvable SIR models, their extensions and their application to sensitive pandemic forecasting. Nonlinear dynamics. 2021;103(3):2955–2971. doi: 10.1007/s11071-021-06248-y 33551570 PMC7849229

[26] Brown EN, Barbieri R, Ventura V, Kass RE, Frank LM. The time-rescaling theorem and its application to neural spike train data analysis. Neural computation. 2002;14(2):325–346. doi: 10.1162/08997660252741149 11802915

[27] Truccolo W, Eden UT, Fellows MR, Donoghue JP, Brown EN. A point process framework for relating neural spiking activity to spiking history, neural ensemble, and extrinsic covariate effects. J Neurophysiol. 2005;93(2):1074–1089. doi: 10.1152/jn.00697.2004 15356183

[28] Vestergaard CL, Génois M. Temporal gillespie algorithm: Fast simulation of contagion processes on time-varying networks. PLoS computational biology. 2015;11(10):e1004579. doi: 10.1371/journal.pcbi.1004579 26517860 PMC4627738

[29] Léonard F, Delamotte B. Critical exponents can be different on the two sides of a transition: A generic mechanism. Physical review letters. 2015;115(20):200601. doi: 10.1103/PhysRevLett.115.200601 26613426

[30] Korniss G, White C, Rikvold P, Novotny M. Dynamic phase transition, universality, and finite-size scaling in the two-dimensional kinetic Ising model in an oscillating field. Physical Review E. 2000;63(1):016120. doi: 10.1103/PhysRevE.63.016120 11304327

[31] Argolo C, Quintino Y, Gleria I, Lyra M. Finite-size scaling analysis of the critical behavior of a general epidemic process in 2D. Physica A: Statistical Mechanics and its Applications. 2011;390(8):1433–1439. doi: 10.1016/j.physa.2010.12.012

[32] Pruessner G. Self-organised criticality: theory, models and characterisation. Cambridge University Press; 2012.

[33] Saggio ML, Crisp D, Scott JM, Karoly P, Kuhlmann L, Nakatani M, et al. A taxonomy of seizure dynamotypes. Elife. 2020;9:e55632. doi: 10.7554/eLife.55632 32691734 PMC7375810

[34] Proix T, Truccolo W, Leguia MG, Tcheng TK, King-Stephens D, Rao VR, et al. Forecasting seizure risk in adults with focal epilepsy: a development and validation study. The Lancet Neurology. 2021;20(2):127–135. doi: 10.1016/S1474-4422(20)30396-3 33341149 PMC7968722

[35] Haken H. Synergetics: An introduction: nonequilibrium phase transitions and self-organization in physics, chemistry, and biology. In: Self-Organizing Systems. Springer; 1987. p. 417–434.

[36] Hinrichsen H. Non-equilibrium critical phenomena and phase transitions into absorbing states. Advances in physics. 2000;49(7):815–958. doi: 10.1080/00018730050198152

[37] Buice MA, Cowan JD. Field-theoretic approach to fluctuation effects in neural networks. Physical Review E. 2007;75(5):051919. doi: 10.1103/PhysRevE.75.051919 17677110

[38] Dorogovtsev SN, Goltsev AV, Mendes JF. Critical phenomena in complex networks. Reviews of Modern Physics. 2008;80(4):1275. doi: 10.1103/RevModPhys.80.1275

[39] Ferreira SC, Castellano C, Pastor-Satorras R. Epidemic thresholds of the susceptible-infected-susceptible model on networks: A comparison of numerical and theoretical results. Physical Review E. 2012;86(4):041125. doi: 10.1103/PhysRevE.86.041125 23214547

[40] Martins P, Plascak J. Probability distribution of the order parameter. Brazilian journal of physics. 2004;34(2A):433–437. doi: 10.1590/S0103-97332004000300021

[41] Tsypin M, Blöte H. Probability distribution of the order parameter for the three-dimensional Ising-model universality class: A high-precision Monte Carlo study. Physical Review E. 2000;62(1):73. doi: 10.1103/PhysRevE.62.73 11088436

[42] Munoz MA. Colloquium: Criticality and dynamical scaling in living systems. Reviews of Modern Physics. 2018;90(3):031001. doi: 10.1103/RevModPhys.90.031001

[43] Bressloff PC. Metastable states and quasicycles in a stochastic Wilson-Cowan model of neuronal population dynamics. Physical Review E. 2010;82(5):051903. doi: 10.1103/PhysRevE.82.051903 21230496

[44] Heck CN, King-Stephens D, Massey AD, Nair DR, Jobst BC, Barkley GL, et al. Two-year seizure reduction in adults with medically intractable partial onset epilepsy treated with responsive neurostimulation: Final results of the RNS System Pivotal trial. Epilepsia. 2014;55(3):432–441. doi: 10.1111/epi.12534 24621228 PMC4233950

[45] El Houssaini K, Bernard C, Jirsa VK. The Epileptor model: a systematic mathematical analysis linked to the dynamics of seizures, refractory status epilepticus and depolarization block. Eneuro. 2020;. 10.1523/ENEURO.0485-18.2019 PMC7096539 32066612

[46] Saggio ML, Spiegler A, Bernard C, Jirsa VK. Fast–Slow Bursters in the Unfolding of a High Codimension Singularity and the Ultra-slow Transitions of Classes. The Journal of Mathematical Neuroscience. 2017;7(1):7. doi: 10.1186/s13408-017-0050-8 28744735 PMC5526832

