# Supplementary material for: Criticality in probabilistic models of spreading dynamics in brain networks: Epileptic seizures
Source: PLoS Comput Biol. 2023 Feb 7;19(2):e1010852. doi: 10.1371/journal.pcbi.1010852 (PMC9904505; doi:10.1371/journal.pcbi.1010852)
Supplement: S1 Text — (PDF) [file pcbi.1010852.s001.pdf]

Criticality in probabilistic models of spreading dynamics in brain networks: epileptic seizures

Supporting Information

S Amin Moosavi[1], Wilson Truccolo[1,2,†]

1 Department of Neuroscience, Brown University, Providence, Rhode Island, USA

2 Carney Institute for Brain Science, Brown University, Providence, Rhode Island, USA

†wilson_truccolo@brown.edu

Structural network connectivity from white-matter tractography

Patient-specific connectivity networks were inferred from white-matter tractography obtained via diffusion MRI [1, 2]. In patient-specific networks P1 - P2, a 84-area (Desikan-Killiany) parcellation of brain areas was used, while for P3-P5 a finer 162-area parcellation was employed.

The inferred patient-specific connectivity matrices $\mathbf{W}$ were further truncated and normalized. To attenuate potentially large artifacts in the estimation of white-matter connectivity, entries larger than the 95% percentile (across all entries) were set to this threshold value. After that, all entries were normalized by this threshold such that $0 \leq W_{ij} \leq 1$. Connectivity matrices for patients P1 - P5 are shown in Fig A.

Time delays τ_{ij} in the model were computed from the length of the estimated white-matter fiber tracts connecting brain areas related to nodes i and j [1]. The maximum length in the 5 different patient-specific connectivity data corresponded to 200 mm. We set the speed to 60 mm per time unit in the model. Given our choice of setting the time unit in the model to 0.02 s (see Methods in the main text for model simulations), a speed of 3000 mm / s (i.e. 60 mm $\times$ 1/0.02s) was obtained, with a maximum time delay of 0.067 s for the largest fiber length of 200 mm. This is slower than expected when compared to only axonal transmission delays, and it should be understood here as including also delays in synaptic activity and rising times in neuronal population responses, for example. We also explored a range of much slower delays, but found no qualitative differences in the spreading dynamics in these Epileptor network models.

We used the same connectivity and time delay matrices previously examined in [3]. The following nodes and brain areas were identified as the epileptogenic zones (EZs)

P1: $\{61, 64\}$ for ctx-rh-lingual and ctx-rh-parahippocampal, respectively;

P2: $\{48, 60, 81\}$ for right-amygdala, ctx-rh-lateral orbito frontal and ctx-rh-temporal pole, respectively;

P3: $\{135, 154, 156\}$ for right-rhinal-cortex, right-hippocampus-anterior and right-amygdala, respectively;

P4: $\{35, 51, 53, 54, 73, 74, 75\}$ for left-temporal-pole, left-collateral-sulcus, left-parahippocampal-cortex, left-rhinal-cortex, left-hippocampus-anterior, left-hippocampus-posterior, left-amygdala, respectively;

P5: $\{83, 87, 116, 126, 135, 154, 156\}$ for right-orbito-frontal-cortex, right-F3-pars-opercularis, right-temporal-pole, right-T2-anterior, right-rhinal-cortex, right-hippocampus-anterior, right-amygdala, respectively.

Here, for purpose of simulation based analyses, we choose only two EZ for each patient-specific network.

References

[1] Timothée Proix, Andreas Spiegler, Michael Schirner, Simon Rothmeier, Petra Ritter, and Viktor K Jirsa. How do parcellation size and short-range connectivity affect dynamics in large-scale brain network models? *NeuroImage*, 142:135–149, 2016.

[2] Timothée Proix, Fabrice Bartolomei, Patrick Chauvel, Christophe Bernard, and Viktor K Jirsa. Permittivity coupling across brain regions determines seizure recruitment in partial epilepsy. *Journal of Neuroscience*, 34(45):15009–15021, 2014.

[3] S Amin Moosavi, Viktor K Jirsa, and Wilson Truccolo. Critical dynamics in the spread of focal epileptic seizures: Network connectivity, neural excitability and phase transitions. *Plos one*, 17(8):e0272902, 2022.

Patient-specific structural connectivity and time delay matrices

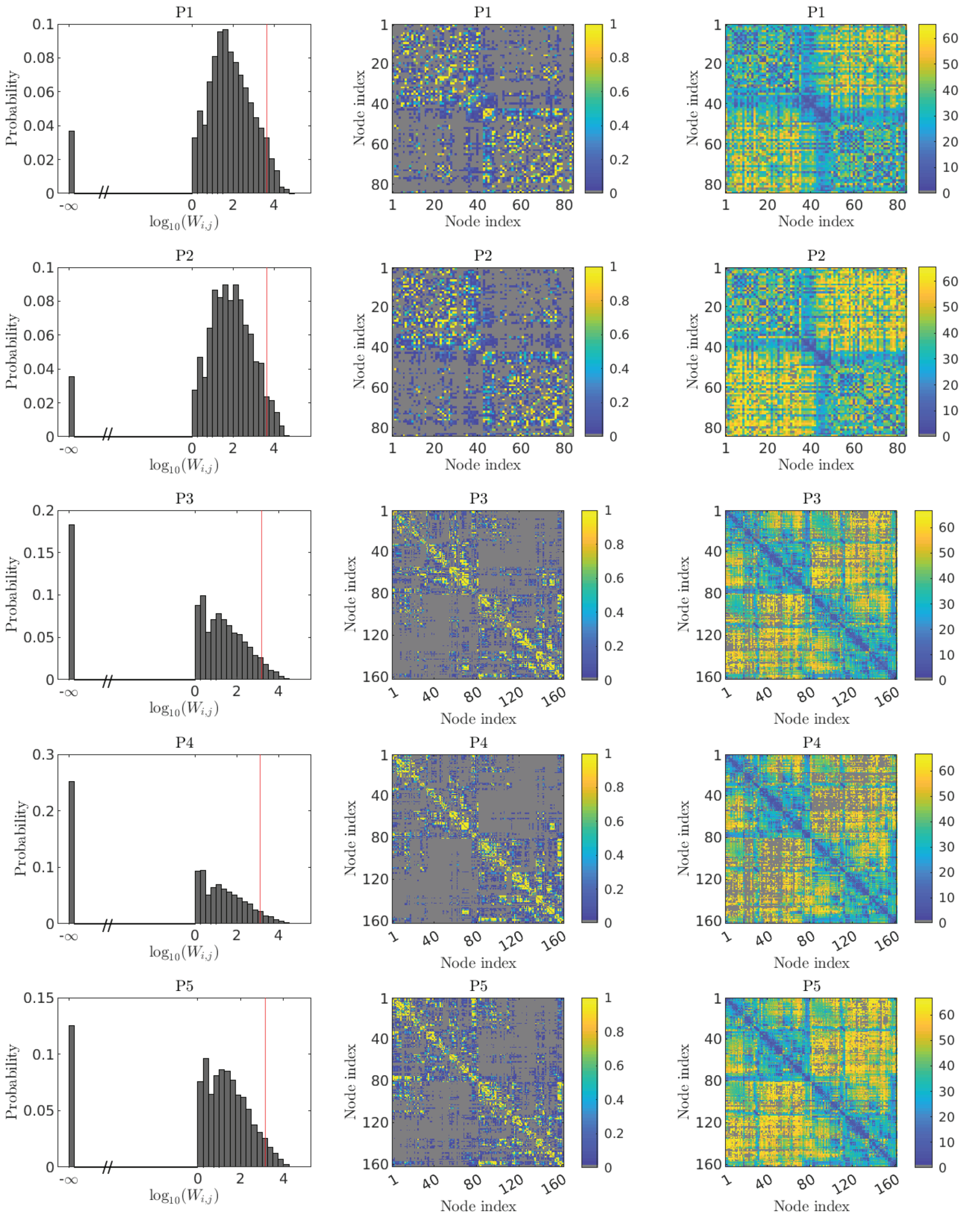

Fig A. **Patient-specific structural connectivity matrices and time delays. (See next page ...)**

(Fig A *continuation*) From top to bottom: patient-specific networks P1 – P5. **Left** Distribution of patient-specific (non-normalized) connectivity weights. The red line indicates the 95% percentile used as a threshold to obtain the truncated and normalized connectivity matrix. **Middle** normalized patient-specific connectivity matrices **W**. **Right** Corresponding propagation time delays τ_{ij} (in milliseconds).

Network	Clustering coefficient	Mean shortest path	Radius	Diameter	Small-world-ness
P1	0.9832	3.3645	5.5939	9.8978	1.6228
P2	0.9834	3.2260	4.6086	8.7336	1.5253
P3	0.9149	2.8358	3.6425	6.6711	1.3248
P4	0.8918	3.0257	4.1177	6.6789	1.3476
P5	0.9375	2.9441	4.0000	6.2852	1.4215

Table A. Graph theoretic measures

In the above table, we used graph theoretic measures defined for weighted networks. For calculation of the shortest path length, the diameter and the radius of the network, the inverse of weights ($L_{i,j} = 1/W_{ij}$) were used. In this way, the shortest path length between two nodes is the minimum sum of the L_{ij} between the two nodes over all the possible paths. Diameter is the maximum of longest paths between all the pairs. Radius is defined as the minimum of the longest paths. The clustering coefficient measures how strongly the nearest neighbors of a node are connected to each other and is calculated as

$$C_i = \frac{1}{(k_i - 1)\sum_j W_{ij}} \sum_{j,k} \frac{W_{ij} + W_{ik}}{2} a_{ij} a_{ik} a_{jk} \tag{1}$$

where $a_{ij} = 1$ if there is a connection from j to i and zero otherwise. Small-world-ness is defined as

$$SWS = \frac{C_R L}{C L_R} \tag{2}$$

where C and L are the average clustering coefficient and the average shortest path length of the network, respectively. C_R and L_R are the corresponding measures obtained from random networks with the same size and weight distribution of as the patient-specific networks used in this study.

Dynamics of the probabilistic model

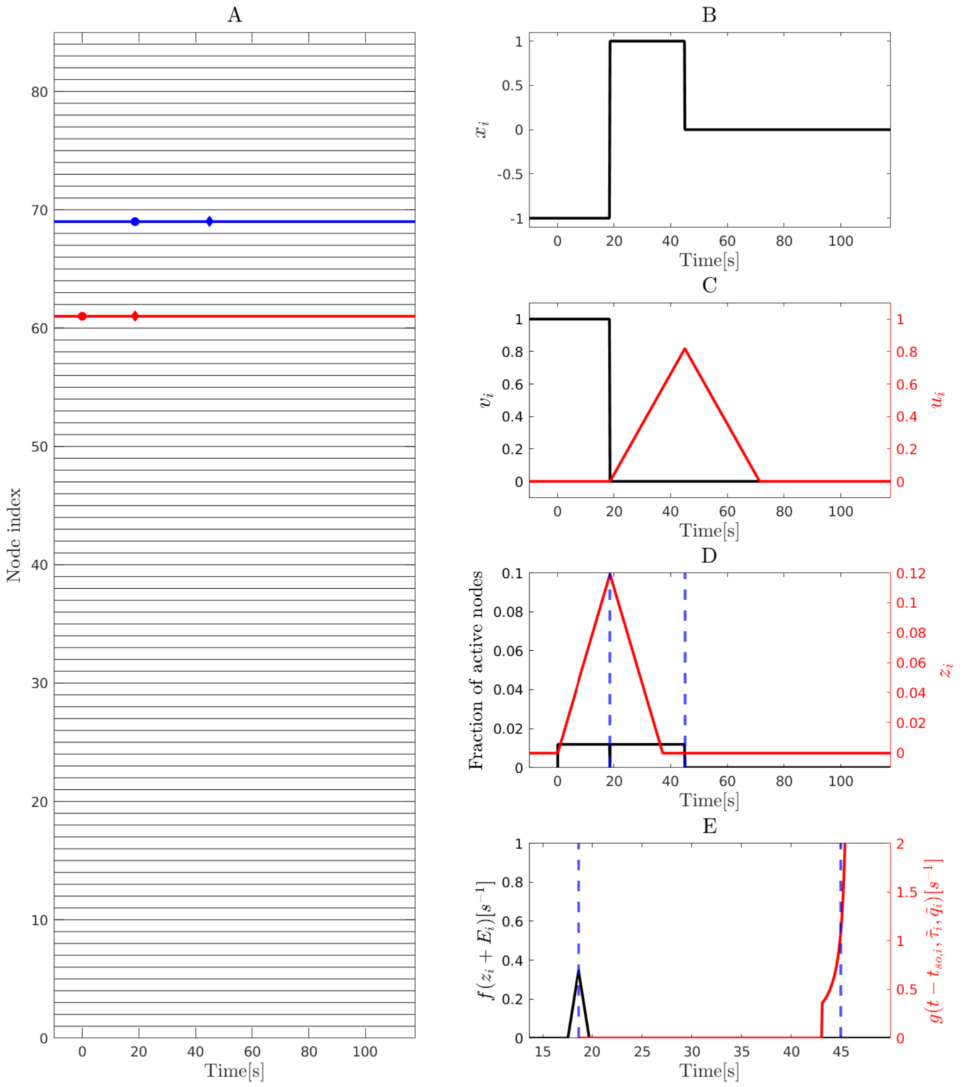

Fig B. **Dynamics of the probabilistic model.** **A** An example of seizure spread dynamics in a patient-specific network (P1) with 84 nodes. Parameters of the model are $a = 0.46$, $b = 0.0021$, $c = 1.3$, $d = 0.05$, $w = 0.45$, $E = -0.112$, $E_{ez} = 0.0026$. Seizure onset and offset times in each node are respectively shown by a circle and a diamond. The seizure starts at the EZ node (red) and spreads to one surrounding node. Panels **B**-**E** show the evolution of different dynamical variables and transition rates of node 69 shown in blue color. **B** Evolution of the dynamical variable x_i. Before seizure initiation $x_i = -1$ (susceptible state), during the seizure $x_i = 1$ (seizure state) and after seizure termination $x_i = 0$ (refractory state). **C** Evolution of variables u_i and v_i defined in Equations 8-9. **D** Evolution of the fraction of active nodes in the system and $z_i(t)$ defined in Equation 7. **E** Evolution of transition rates $f(z_i + E_i)$ for transition from susceptible to seizure and $g(t - t_{so}, \tilde{\tau}_{s,i}, \tilde{q}_{s,i})$ for transition from seizure to refractory state. Blue dashed lines indicate seizure onset and offset times.

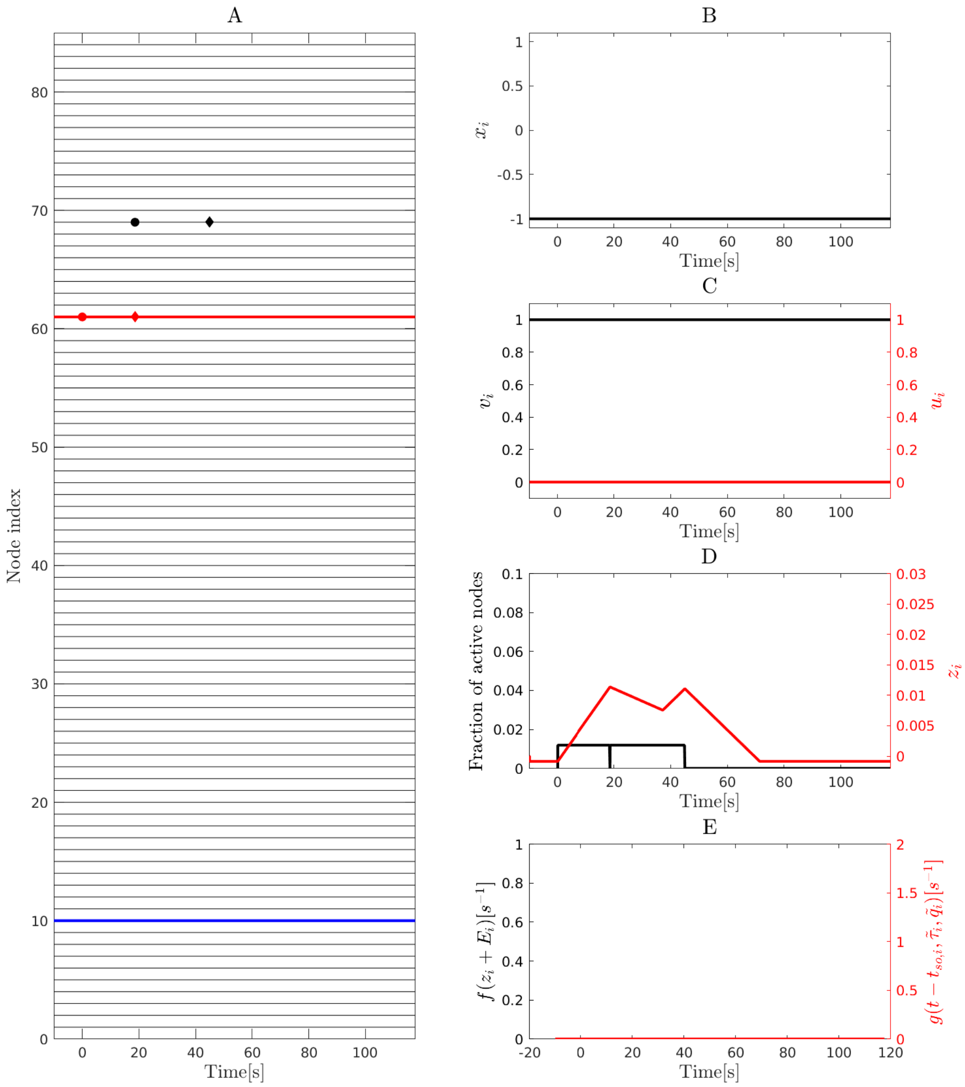

Fig C. **Dynamics of the probabilistic model. A** An example of seizure spread dynamics in a patient-specific network (P1) with 84 nodes. Parameters of the model are $a = 0.46$, $b = 0.0021$, $c = 1.3$, $d = 0.05$, $w = 0.45$, $E = -0.112$, $E_{ez} = 0.0026$. Seizure onset and offset times in each node are respectively shown by a circle and a diamond. The seizure starts at the EZ node (red) and spreads to one surrounding node. Panels **B-E** show the evolution of different dynamical variables and transition rates of node 10 shown in blue color. **B** Evolution of the dynamical variable x_i. Since there is no transition $x_i = -1$ at all times (susceptible state). **C** Variables $u_i = 0$ and $v_i = 1$ defined in Equations 8-9 are constants for this node. **D** Evolution of the fraction of active nodes in the system (black) and $z_i(t)$ (red) defined in Equation 7. **E** Transition rates $f(z_i + E_i)$ for transition from susceptible to seizure and $g(t - t_{so}, \tilde{\tau}_{s,i}, \tilde{q}_{s,i})$ are both equal to zero for this node.

Comparison of phase diagrams

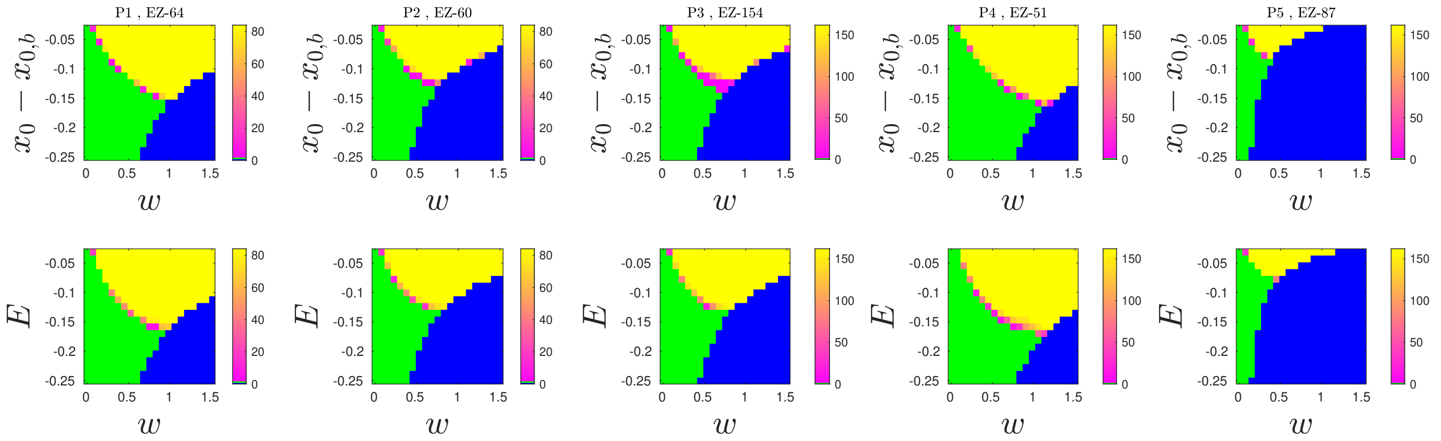

Fig D. **A comparison of the phase diagrams of patient-specific Epileptor network models (top row) and from the proposed model (bottom row) for 5 different patient-specific networks.** In contrast to the Fig 2 in the main text, here the phase diagrams refer to a different EZ. Same conventions as in Fig 2, main text

Comparison of onset-times (spread ranks)

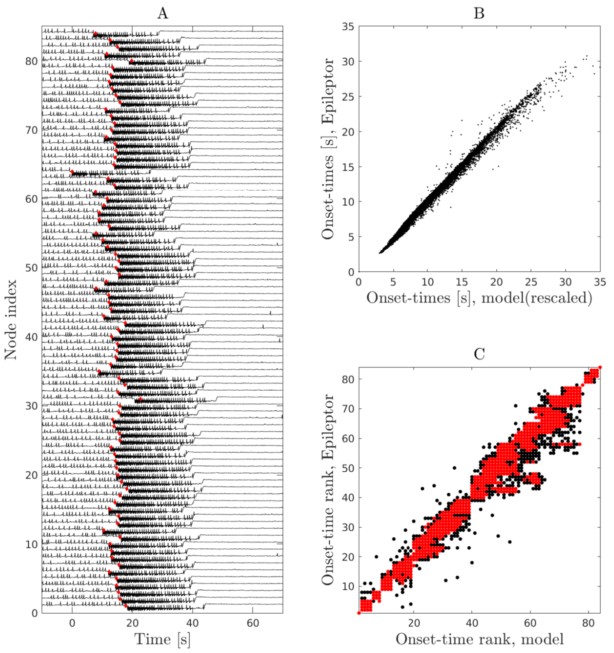

Fig E. **The proposed model captures the spread timing in patient-specific Epileptor network models: example from P1, EZ-64.**

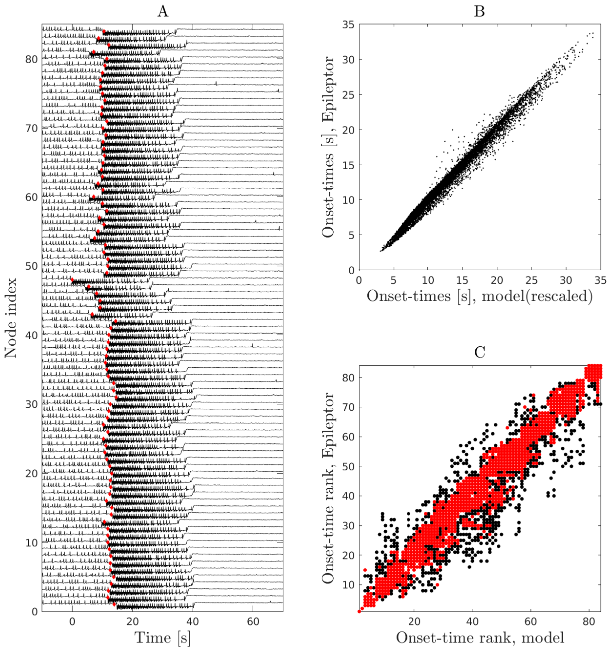

Fig F. **The proposed model captures the spread timing in patient-specific Epileptor network models: example from P2, EZ-48.**

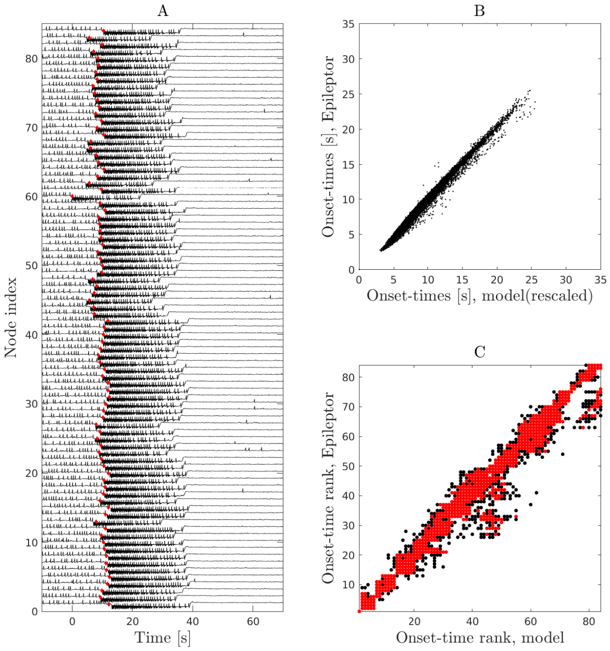

Fig G. **The proposed model captures the spread timing in patient-specific Epileptor network models: example from P2, EZ-60.**

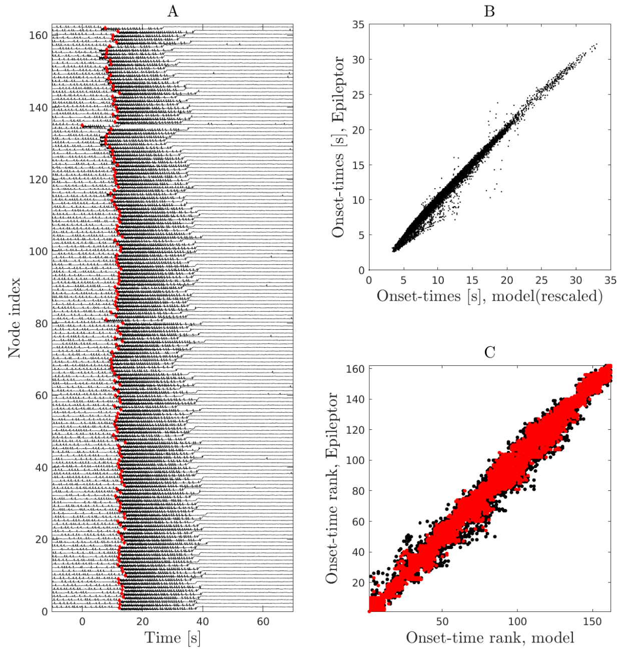

Fig H. **The proposed model captures the spread timing in patient-specific Epileptor network models: example from P3, EZ-135.**

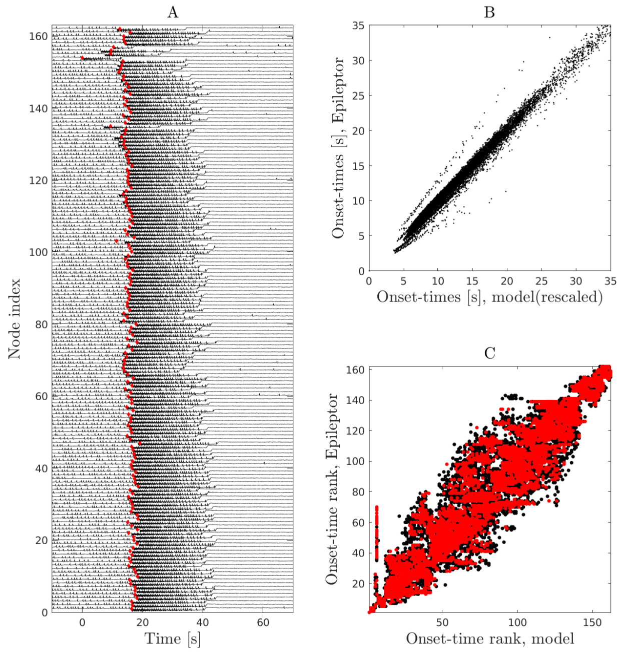

Fig I. **The proposed model captures the spread timing in patient-specific Epileptor network models: example from P3, EZ-154.**

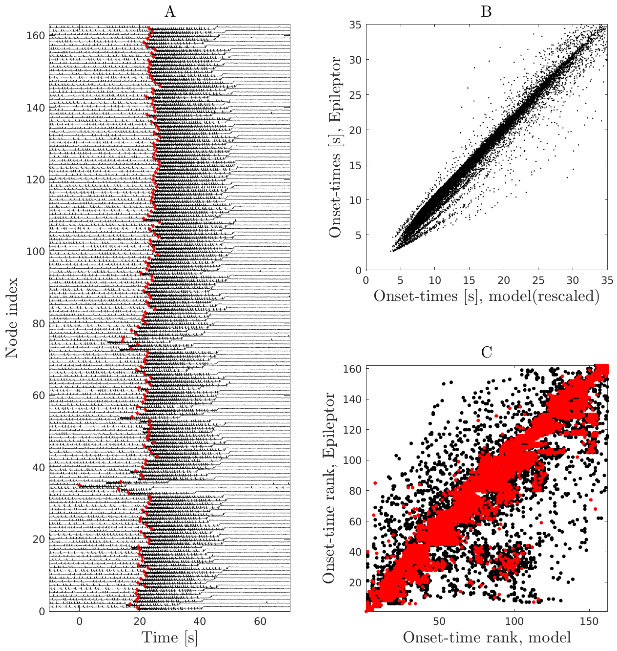

Fig J. **The proposed model captures the spread timing in patient-specific Epileptor network models: example from P4, EZ-35.**

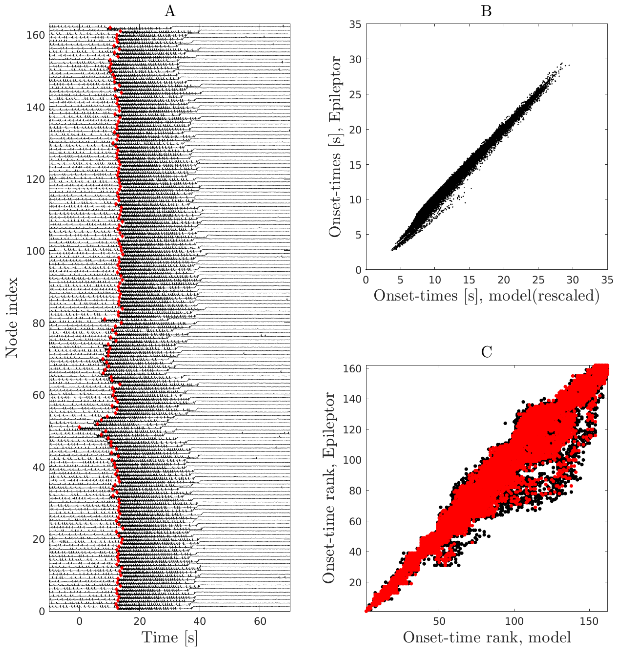

Fig K. **The proposed model captures the spread timing in patient-specific Epileptor network models: example from P4, EZ-51.**

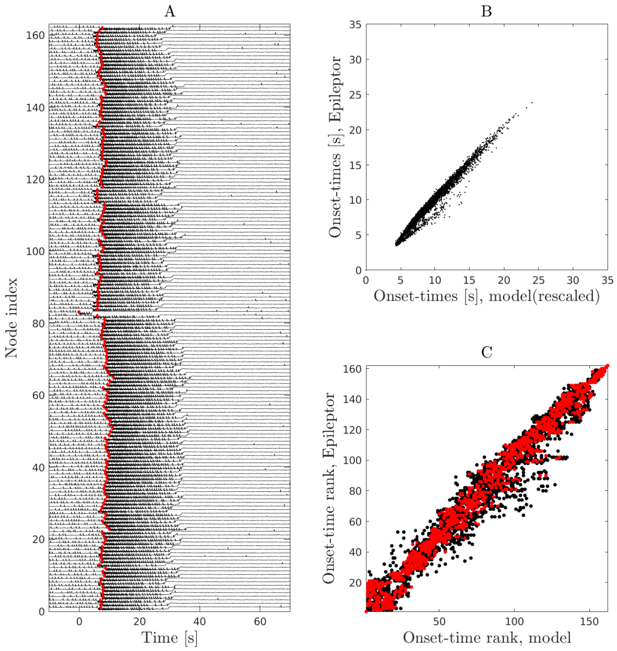

Fig L. **The proposed model captures the spread timing in patient-specific Epileptor network models: example from P5, EZ-83.**

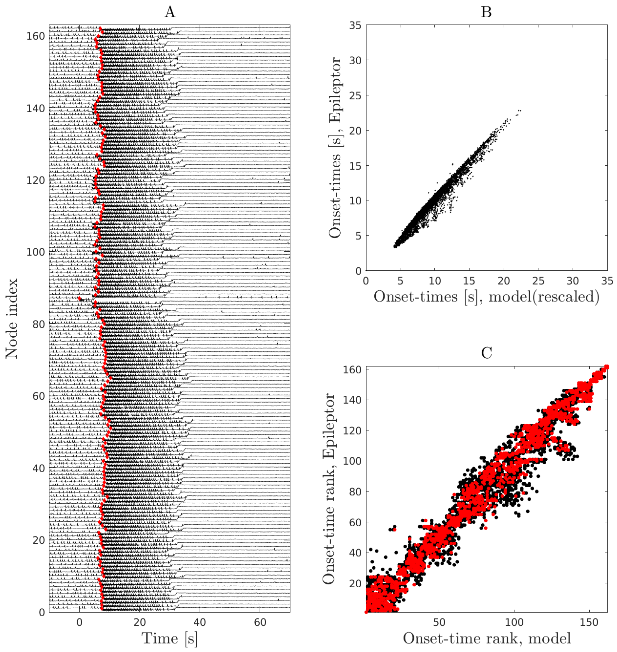

Fig M. **The proposed model captures the spread timing in patient-specific Epileptor network models: example from P5, EZ-87.**

Temporal evolution of spreading dynamics

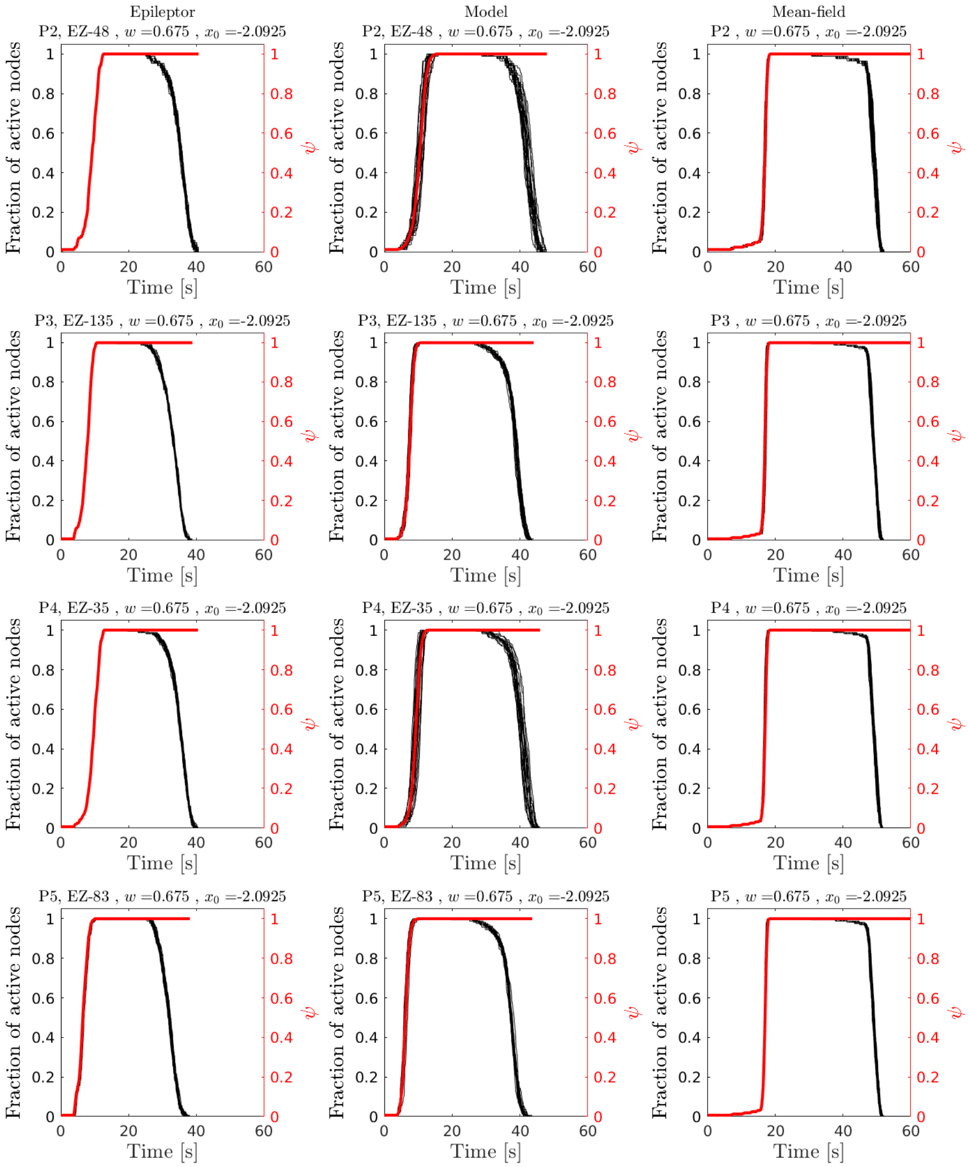

Fig N. **Temporal evolution of spread size and fraction of active (seizing) nodes: examples for the four other patient-specific networks.** Same conventions as in Fig 4, main text.

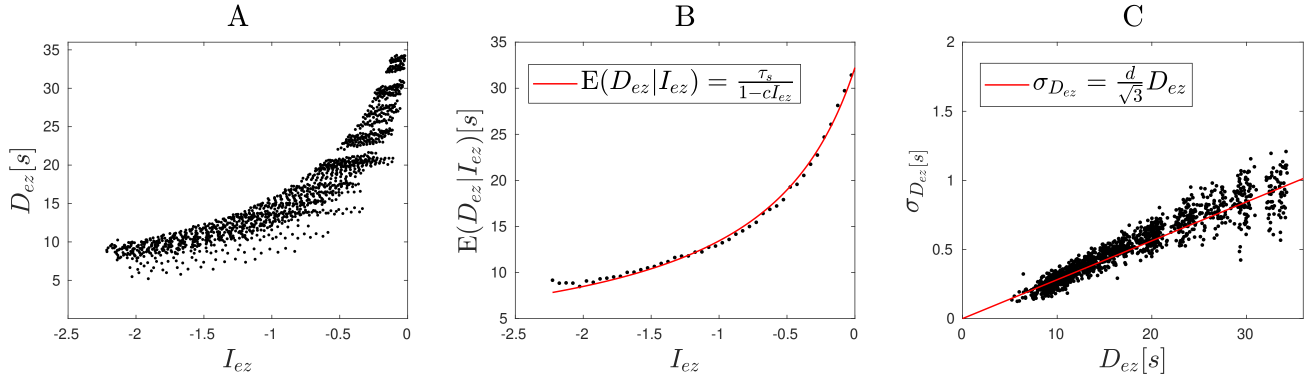

Fig O. **Fitting of the proposed model to patient-specific Epileptor networks: exploiting the dependence on seizure duration in the EZ node. A** The duration of seizures in EZ node as a function to its input from the surrounding nodes, i.e. $I_{ez} = wE\sum_i W_{ez,i}$. **BC** Fitting of the corresponding expected value $E(D_{ez}|I)$ and standard deviation $\sigma_{D_{ez}}$, respectively, results in $\tau_s = 32.22$ s, $c = 1.3$ and $d = 0.05$.

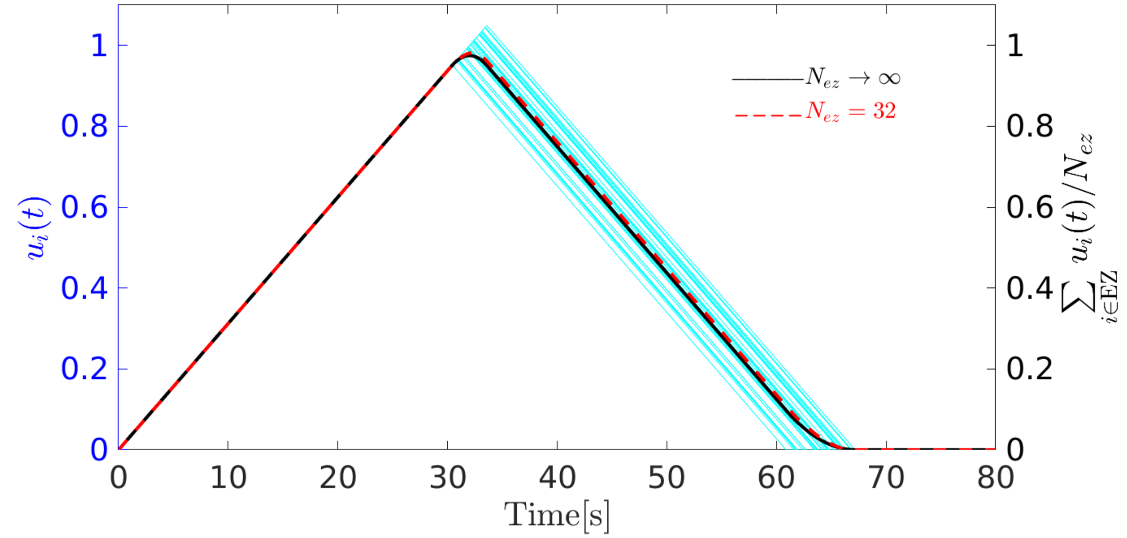

Fig P. **The total output of EZ nodes when all the EZ nodes go to seizure at $t = 0$ exhibits a maximum point.** The cyan color shows $u_i(t)$ of 32 EZ nodes that simultaneously go to seizure at time zero. Seizures start to terminate at 30 seconds. Seizures in other node terminate between 30 to 34 seconds according to a uniform distribution. Red dashed curve shows the average of $u_i(t)$ across all 32 EZ nodes. The black curve is the large N_{ez} approximation of the average of $u_i(t)$ ($U(t)$ defined in Equation 42). Note that this average is proportional to the total output of the EZ nodes in the mean-field approximation.
